# Rapid RGR-dependent visual pigment recycling is mediated by the RPE and specialized Müller glia

**DOI:** 10.1016/j.celrep.2023.112982

**Published:** 2023-08-15

**Authors:** Aleksander Tworak, Alexander V. Kolesnikov, John D. Hong, Elliot H. Choi, Jennings C. Luu, Grazyna Palczewska, Zhiqian Dong, Dominik Lewandowski, Matthew J. Brooks, Laura Campello, Anand Swaroop, Philip D. Kiser, Vladimir J. Kefalov, Krzysztof Palczewski

**Affiliations:** 1 Department of Ophthalmology, Gavin Herbert Eye Institute, University of California, Irvine, Irvine, CA 92697, USA; 2 Department of Pharmacology, Case Western Reserve University, Cleveland, OH 44106, USA; 3 Polgenix, Inc., Department of Medical Devices, Cleveland, OH 44106, USA; 4 Neurobiology, Neurodegeneration and Repair Laboratory, National Eye Institute, National Institutes of Health, Bethesda, MD 20892, USA; 5 Department of Physiology & Biophysics, University of California, Irvine, Irvine, CA 92697, USA; 6 Department of Clinical Pharmacy Practice, University of California, Irvine, Irvine, CA 92697, USA; 7 Research Service, VA Long Beach Healthcare System, Long Beach, CA 90822, USA; 8 Department of Chemistry, University of California, Irvine, Irvine, CA 92697, USA; 9 Department of Molecular Biology and Biochemistry, University of California, Irvine, Irvine, CA 92697, USA; 10 Lead contact

## Abstract

In daylight, demand for visual chromophore (11-*cis*-retinal) exceeds supply by the classical visual cycle. This shortfall is compensated, in part, by the retinal G-protein-coupled receptor (RGR) photoisomerase, which is expressed in both the retinal pigment epithelium (RPE) and in Müller cells. The relative contributions of these two cellular pools of RGR to the maintenance of photoreceptor light responses are not known. Here, we use a cell-specific gene reactivation approach to elucidate the kinetics of RGR-mediated recovery of photoreceptor responses following light exposure. Electroretinographic measurements in mice with RGR expression limited to either cell type reveal that the RPE and a specialized subset of Müller glia contribute both to scotopic and photopic function. We demonstrate that 11-*cis*-retinal formed through photoisomerization is rapidly hydrolyzed, consistent with its role in a rapid visual pigment regeneration process. Our study shows that RGR provides a pan-retinal sink for all-*trans*-retinal released under sustained light conditions and supports rapid chromophore regeneration through the photic visual cycle.

## In brief

Tworak et al. report that the RGR-mediated photic visual cycle found in the retinal pigment epithelium and in specialized Müller glia in the mammalian retina constitutes a fast visual-pigment recycling pathway that modulates both cone function in bright light and rod dark adaptation upon the transition to darkness.

## Graphical Abstract

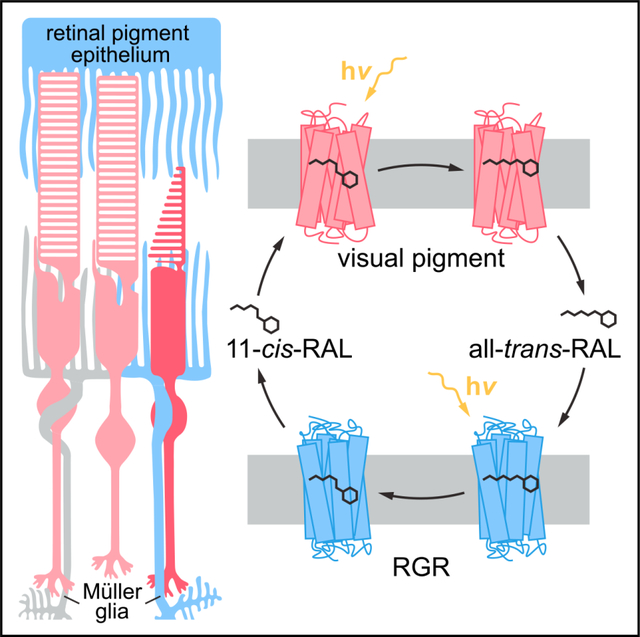

## INTRODUCTION

Mammalian vision is mediated mainly by two types of photoreceptors: rods, which enable vision in dim light, and cones, which function in bright light and support color discrimination. Both cell types contain visual pigments composed of an opsin protein, covalently bound to an 11-*cis*-retinylidene chromophore via a Schiff base linkage to a Lys residue side chain. Absorption of a photon causes *cis-trans* isomerization around the C^11^–C^12^ double bond of the chromophore to activate the visual pigment, followed by hydrolysis of the Schiff base and eventual release of all-*trans*-retinal. The released all-*trans*-retinal must be recycled to the 11-*cis* configuration to regenerate and maintain a high abundance of visual pigments sustaining light sensitivity and continuous vision. 11-*cis*-retinoids are higher in free energy than their all-*trans* isomers due to steric interactions between the C^10^-H and C^13^-methyl groups,^1^ and the *trans-cis* isomerization involves kinetically unfavorable molecular rotations around the polyene backbone. Thus, recovery of the less thermodynamically stable 11-*cis* configuration must occur through enzymatic activity or at the expense of energy afforded by the absorption of light quanta.

Two enzymes abundant in the mammalian eyes are capable of catalyzing the *trans-cis* retinoid isomerization required for the regeneration of visual pigment: retinoid isomerase (RPE65) and retinal G-protein-coupled receptor (RGR).^2^ RPE65, a 65-kDa protein expressed in the retinal pigment epithelium (RPE), acts as a crucial component of the classical visual cycle.^3^ This process, occurring across the RPE and photoreceptor cells, involves a series of light-independent enzymatic reactions that produce 11-*cis*-retinal in quantities sufficient to support rod function yet insufficient to maintain cone photosensitivity under normal daylight conditions.^4^ Numerous studies have established the important role of Müller glia in supplying additional active chromophore to specifically support cone functionality.^5^ The only Müller glia *trans-cis* retinoid isomerase identified to date is RGR.^6,7^

RGR belongs to the opsin family, but it exhibits weaker sequence conservation compared with its other members.^8^ It is expressed in the RPE and in Müller glia of most mammals, including human, cow, and mouse, with the notable exception of mostly nocturnal marsupials.^8,9^ Compared with other G protein-coupled receptors (GPCRs), mammalian RGR proteins contain several amino acid substitutions in highly conserved regions involved in receptor activation; these mutations likely abolish GPCR signaling capability of the mammalian RGRs.^8^ However, a conserved feature shared by RGR opsins is the presence of a Lys residue in transmembrane helix 7, which preferentially binds all-trans-retinal through a covalent Schiff base linkage and is crucial for the enzyme’s *trans-cis* photoisomerase activity.^9,10^ This process is optimal with stimulation by 530-nm light, corresponding to RGR action spectrum maximum, and in the presence of cellular retinaldehyde-binding protein (CRALBP).^9^ Since CRALBP, like RGR, is expressed in the RPE and in Müller glia in mammalian retinas, both cell types are well primed to support photic visual pigment regeneration catalyzed by RGR. However, the relative contributions of the RPE and the Müller glia to RGR-mediated visual pigment regeneration and whether those two pools of RGR have distinct functional roles in the retina remain unknown.

Under bright light conditions, rod contribution to visual perception is obfuscated by physiological saturation; however, their visual pigment still undergoes continuous bleaching and regeneration cycles, maintaining a high demand for 11-*cis*-retinal. Cones, primarily involved in photopic vision, compete with rods for the chromophore supply,^11^ which has been shown to limit the dark adaptation rate for both photoreceptor types.^12^ Nevertheless, visual pigment regeneration occurs faster in cones than in rods, suggesting that some source of 11-*cis*-retinal preferentially supports cone functionality.^13,14^ Previous studies have demonstrated that a global loss of *Rgr* in mice decreases the rate of 11-*cis*-retinal synthesis under sustained light exposure^15^ and that RGR activity contributes to cone photoreceptor sensitivity in daylight conditions.^16^ The current study was designed to establish the kinetics of RGR-mediated chromophore production, to analyze the distribution of RGR between the RPE and Müller glia in different species, and to determine the relative contributions of the two distinct RGR pools to rod and cone function.

## RESULTS

### RGR facilitates rapid photoproduction of visual chromophore in native membranes

**Figure 1.**
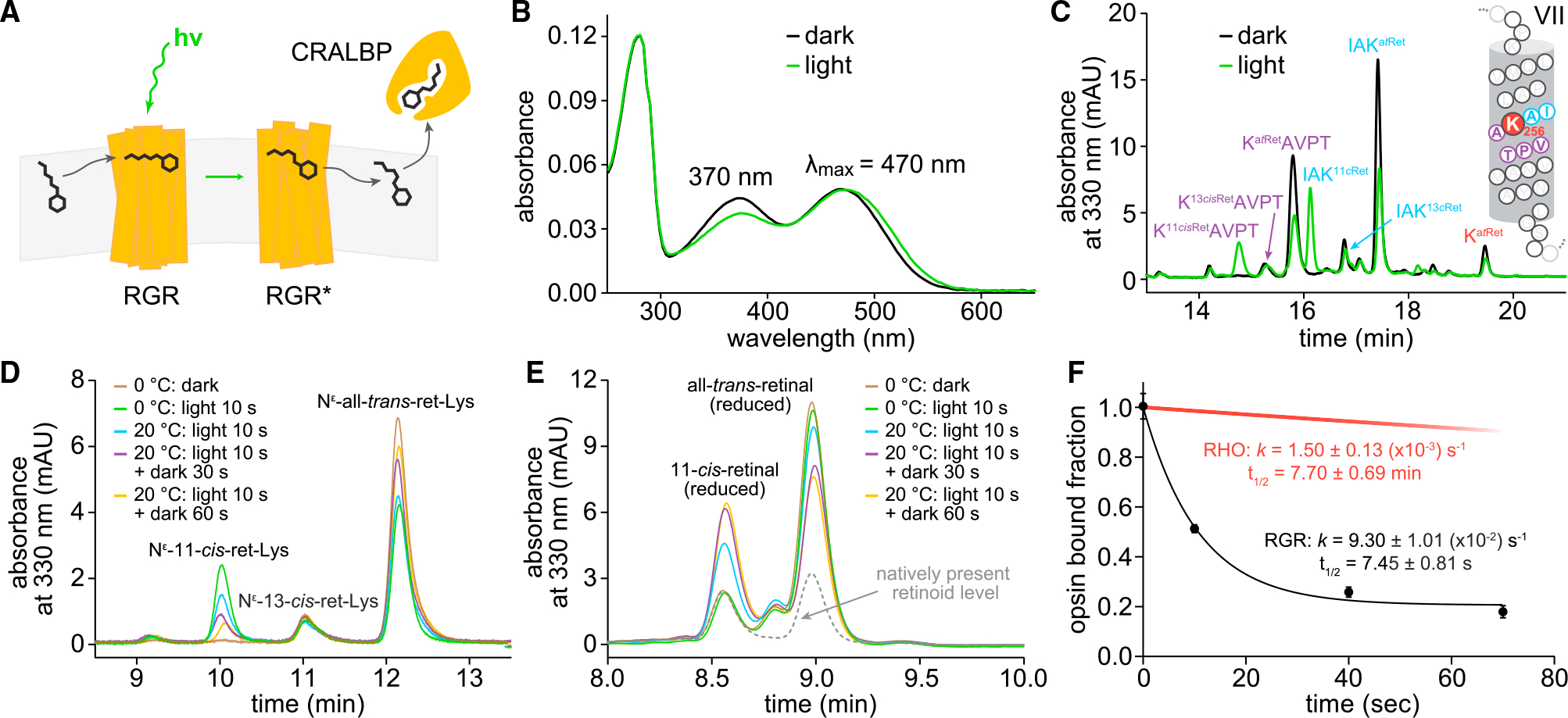
RGR* hydrolysis facilitates rapid production of 11-*cis*-retinal in native microsomal membranes (A) Scheme of RGR turnover of all-*trans*-retinal to 11-*cis*-retinal under light. (B) UV-vis absorbance spectra of purified bovine RGR regenerated with all-*trans*-retinal solubilized in LMNG, showing minor spectral changes after light exposure on ice. (C) Chromatographic separation and mass spectroscopic identification of N^ε^-retinyl-peptides of RGR, from proteinase K digestion of sodium borohydrate (NaBH_4_)/isopropanol (*i*PrOH)-treated RPE microsomes, before and after light exposure. N^ε^-retinyl-peptide products reflect all-*trans*-retinylidene Schiff base adducted to Lys^256^ of RGR. For detailed LC-MS/MS analyses, see Figure S1. (D) Pronase digestion of RPE microsomal fraction enables direct measurement of photoisomerization of the RGR all-*trans*-retinylidene adduct to the 11-*cis* configuration, followed by hydrolysis of the 11-*cis*-retinylidene Schiff base, accompanied by corresponding regeneration of the RGR over time by readduction of new all-*trans*-retinal. For detailed LC-MS/MS analyses, see Figure S2. (E) Retinoid analysis of lipid-soluble fraction shows reciprocal increase in 11-*cis*-retinal production with hydrolysis of RGR* 11-*cis*-retinylidene Schiff base over time. The slight excess of exogenous all-*trans*-retinal initially used to produce RGR photopigment in microsomal membranes began decreasing as RGR* hydrolysis freed Lys^256^ to form new all-*trans*-retinylidene Schiff base. (F) Time course of RGR*-11-*cis*-retinylidene Schiff base hydrolysis producing 11-*cis*-retinal. Pseudo-first-order kinetics were observed. Values are plotted as mean ± SD; n = 4 independent experiments. Red line indicates RHO* hydrolysis rate measured previously.^19^

RGR reacts with all-*trans*-retinal, forming the all-*trans*-retinylidene Schiff-base adduct that photoisomerizes to the 11-*cis* configuration, and is hydrolyzed to release 11-*cis*-retinal (Figure 1A). The photoproduction of 11-*cis*-retinal by RGR involves retinylidene photoisomerization, occurring in the timescale of femtoseconds,^17,18^ followed by hydrolysis of the Schiff base, yielding the 11-*cis* chromophore at a rate that has remained unknown. Since hydrolysis occurring with retinylidene proteins differ significantly between native membranes and detergent micelles of purified protein,^19^ we decided to study the kinetics of RGR turnover in native microsomes isolated from bovine RPE cells. The photoreactive all-*trans*-retinylidene pigment of RGR displayed a broad UV-visible (UV-vis) absorbance spectrum with characteristic 470- and 370-nm maxima (Figure 1B).^9^ Following a 10-s exposure to light at the 530-nm action spectrum maximum, the *trans-cis* photoisomerization, which was not readily distinguishable via absorption spectroscopy, was detected by liquid chromatography-tandem mass spectrometry (LC-MS/MS), showing the appearance of N^ε^-11-*cis*-retinyl-peptides after proteolysis by proteinase K (Figures 1C and S1) or pronase (Figures 1D and S2). To study the hydrolysis kinetics, we first illuminated the all-*trans*-retinal-supplemented RPE microsomes for 10 s on ice, aiming to maximize 11-*cis*-retinylidene formation while minimizing its hydrolysis. Indeed, we observed no change from baseline amounts of free 11-*cis*-retinal after this step (Figure 1E). To allow for hydrolysis to proceed, the samples were warmed to 20°C and further illuminated for 10 s, at which point a significant amount of hydrolysis (~50%) was already observed. Afterward, the hydrolysis proceeded in the dark for up to a minute as evidenced by the progressive diminution of the 11-*cis*-retinyl-Lys signal (Figure 1D) and the corresponding increase in free 11-*cis*-retinal (Figure 1E). Concurrent with the hydrolysis of the 11-*cis*-retinylidene moiety to the RGR photoproduct (RGR*) and the release of 11-*cis*-retinal from the chromophore-binding pocket, RGR was undergoing regeneration by all-*trans*-retinal as shown by the increase in all-*trans*-retinyl-Lys signal (Figure 1D) and the corresponding decrease in free all-*trans*-retinal (Figure 1E). The rate of hydrolysis of the 11-*cis*-retinylidene Schiff base of RGR* was on the order of seconds, with a half-life of 7.5 s at 20°C. This hydrolysis rate for RGR* was nearly 60 times faster than that of the all-*trans*-retinylidene Schiff base of photoactivated rhodopsin (RHO*) at 20°C, measured previously (Figure 1F).^19^ Overall, the rapid hydrolysis of RGR* to produce 11-*cis*-retinal and apo-RGR, and the concurrent reformation of all-*trans*-retinylidene-RGR with available all-*trans*-retinal, demonstrates the capacity of RGR to process high quantities of bleached chromophore and support its rapid recycling under photic conditions.

### Specialized Müller glia support chromophore photoproduction

**Figure 2.**
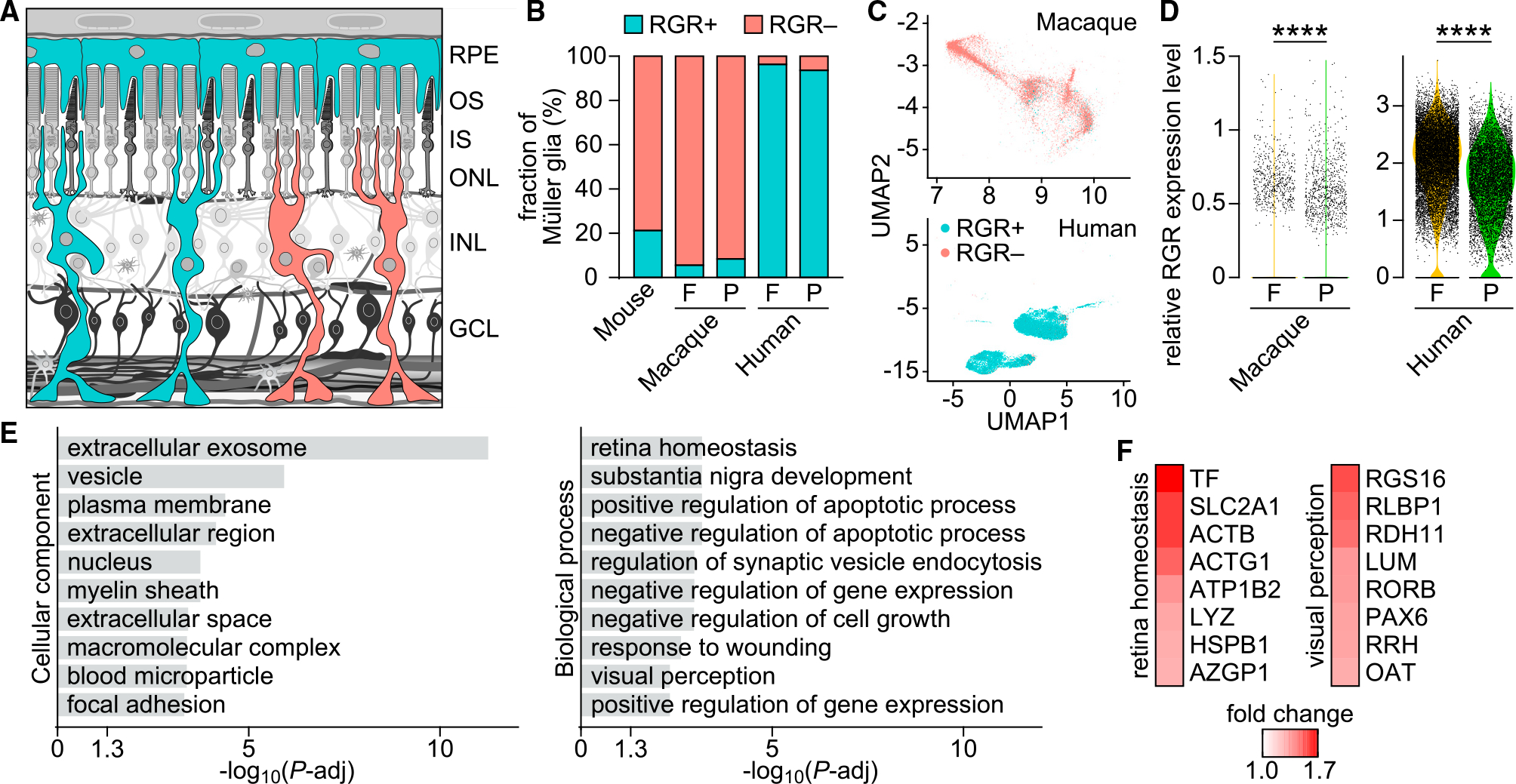
Subpopulation of Müller glia expresses RGR in mammals (A) Cross-sectional diagram of retina indicating its major layers: RPE cells (RPEs), photoreceptor outer segments (OSs), photoreceptor inner segments (ISs), outer nuclear layer (ONL), inner nuclear layer (INL), and ganglion cell layer (GCL). Sites of RGR expression (RPEs and Müller glia) are highlighted in teal. Mammalian retinas contain two subpopulations of Müller glia, characterized by the presence (+) or the absence (−) of RGR. (B) Analysis of proportions of RGR+ and RGR− Müller glia in mouse (n = 1,395 cells), macaque (n = 14,674 cells),^21^ and human (n = 21,066 cells).^22^ In primates, foveal (F) and peripheral (P) retina samples were analyzed separately. (C) UMAP plot showing independent clustering of Müller glia from primate retinas, based on data from (B). The RGR expression status is indicated in color. (D) Violin plots showing the expression level of RGR in F and P Müller glia from macaque and human, based on data from (B). ****p < 0.0001 by Student’s t tests. (E) List of top 10 cellular component- and biological process-GO terms significantly enriched (adjusted p-value, [p-adj] < 0.01) in macaque DEGs (>1.25-fold change) between RGR+ and RGR− Müller glia. p-adj values were calculated using Benjamini-Hochberg method. (F) Heatmaps showing fold change of retina homeostasis- and visual perception-related DEGs significantly enriched in the dataset.

The overall ocular distribution of RGR is conserved across various mammals and confined to the RPE and Müller glia cells.^6,9,16,20^ Notable differences were, however, observed with murine Müller glia, showing significantly lower expression of RGR compared with other species.^9,16^ To better understand species-specific differences in the RGR transcriptional landscapes, we performed a comparative analysis of public single-cell RNA sequencing (scRNA-seq) data from mouse, macaque, and human. In mouse, we obtained 71,632 high-quality transcriptomes, including 1,395 classified as Müller glia based on expression of known markers of this cell type.^21^ In this cohort, at the level of the individual cells, we detected Müller glia characterized either by the presence (+) or the absence (−) of RGR expression (Figure 2A), with RGR+ cells constituting only 21.2% of the total fraction (Figure 2B). For comparison, we analyzed 14,574 and 21,066 Müller glial transcriptomes, obtained from macaque^21^ and human^22^ scRNA-seq datasets, respectively. Here, the fraction of RGR+ Müller glia varied significantly between species: from relatively small proportions in macaque (5.5% in fovea, 8.4% in periphery) to very high in human (96.3% in fovea, 93.6% in periphery) (Figure 2B). Intriguingly, uniform manifold approximation and projection (UMAP) analyses segregated the Müller glia into two distinct clusters, particularly in the human context, corresponding to their foveal or peripheral origin (Figure 2C). Comparison of region-specific data showed elevated average expression of RGR in the foveal Müller glia of both species (Figure 2D).

Next, we compared transcriptomic profiles of RGR+ and RGR− cells to identify differentially expressed genes (DEGs) associated with the RGR status. The analysis was performed on the macaque dataset, owing to its high number of Müller glial transcriptomes compared with mouse and its minimal post-mortem delay times compared with human samples. We observed significant differential expression (adjusted p value [p-adj] < 0.01) of 2,088 genes up to a 1.7-fold change between the RGR+ and RGR− cells. To date, no distinct subtypes of Müller glia have been identified; however, it is conceivable, given these findings, that some level of transcriptional fine-tuning distinguishes the RGR+ Müller glia with optimized photic chromophore regeneration. To further investigate this idea, we performed Gene Ontology (GO) term enrichment analysis of the DEGs associated with the Müller glial RGR expression status in macaque. We found significant over-representation of proteins localized to the extracellular exosomes, suggesting the potential role of exosome release by RGR+ Müller glia in photic visual pigment regeneration. The top enriched biological processes included terms related to retina homeostasis and transcriptional regulation, as well as to visual perception (Figure 2E). The observed combinatorial elevation of multiple transcription factors, including lumican (*LUM*), nuclear receptor ROR-beta (*RORB*), and paired box protein PAX-6 (*PAX6*), well known for their association with retina development, could underlie a mechanism by which the subpopulation of RGR+ Müller glia acquire and maintain the capacity to support photic visual pigment regeneration. Notably, two observed differences pertinent to the photic visual cycle included elevated expression (~1.4-fold) of CRALBP (*RLBP1*) and retinol dehydrogenase 11 (*RDH11*) in RGR+ cells (Figure 2F). The presence of CRALBP is known to strongly promote 11-*cis*-retinal production by RGR, likely by protecting the chromophore from loss by the thermal reisomerization.^9^ RDH11 exhibits the highest expression among all RDH enzymes present in human or macaque Müller glia (Figure S3A), and its dual-substrate specificity allows it to catalyze oxidation of either all-*trans*- or *cis*-retinols to their aldehyde forms in the presence of NADP^+^.^23^ Finally, RGR+ cells showed elevated (~1.5-fold) levels of the major glucose transporter GLUT1 (*SLC2A1*), which can reflect the increased energy demand associated with the photic visual pigment regeneration. Altogether, these findings support the conclusion that the RGR+ subpopulation of Müller glia can be distinguished by physiologically relevant, coordinated transcriptomic differences that facilitate photic visual pigment regeneration.

### Loss of RGR leads to altered retinoid metabolism, preventing all-*trans*-retinal accumulation

**Figure 3.**
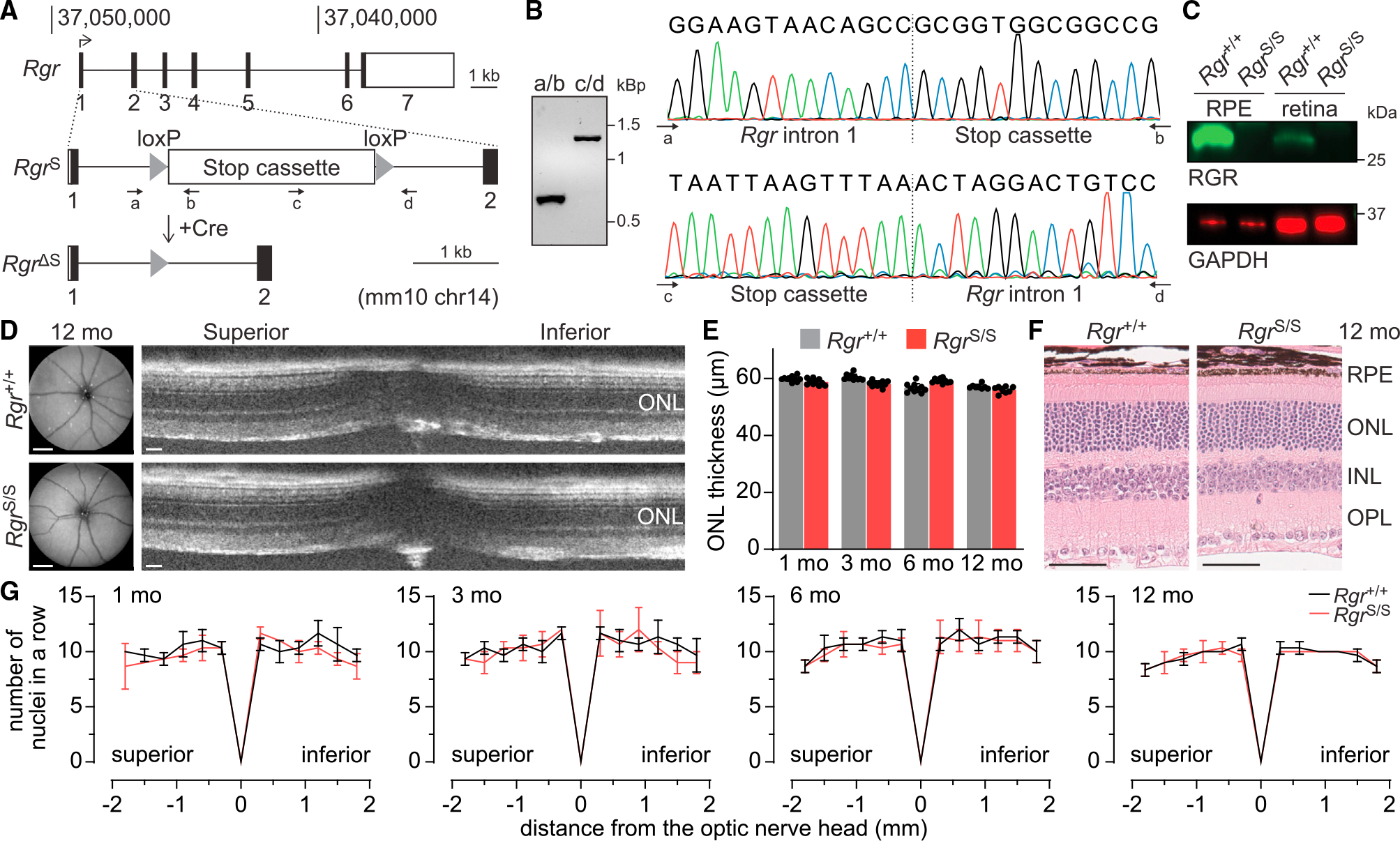
General characteristics of *Rgr*^S^ mice (A) Generation of the conditional rescue of the *Rgr* mouse model. A stop cassette flanked by *loxP* sites was introduced into *Rgr* intron 1. The cassette can be removed by Cre recombinase. (B) Stop cassette integration sites amplified from the genomic DNA by PCR with primer pairs a/b and c/d (indicated in A) and verified by Sanger sequencing. (C) RGR level in protein extracts from retinal and RPE-eyecup preparations from *Rgr*^+/+^ and *Rgr*^S/S^ mice; immunoblotting confirmed successful knockout of RGR upon introduction of the stop cassette. GAPDH served as a loading control. Shown are images representative of n = 3 independent experiments. (D) SLO (left, scale bars: 500 μm) and retinal OCT (right, scale bars: 50 μm) images of 12-month-old *Rgr*^+/+^ and *Rgr*^S/S^ animals, showing no features that distinguish the two lines. (E) OCT-based quantification of ONL thickness in 1-, 3-, 6-, and 12-month-old *Rgr*^+/+^ and *Rgr*^S/S^ mice, measured 500 mm from the ONH; data are shown as mean ± SEM, n = 10 eyes (1, 3, and 6 months old), n = 8 eyes (12 months old). (F) H&E-stained retinal sections of 12-month-old *Rgr*^+/+^ and *Rgr*^S/S^ animals showing no signs of structural retinal pathology upon RGR loss. Images were taken approx. 500 μm from the ONH. Scale bars: 50 μm. (G) Number of nuclei per row quantified in histological sections along the superior-inferior axis of the eyes from 1-, 3-, 6-, and 12-month-old *Rgr*^+/+^ and *Rgr*^S/S^ mice. Data are shown as mean ± SEM; n = 3 eyes.

To enable detailed studies on the role of RGR in sustaining vision, we generated a transgenic mouse allele *Rgr*^Stop^ (*Rgr*^S^) whereby expression of native *Rgr* is blocked by a transcriptional stop cassette, flanked by the *loxP* sites, and excisable by Cre recombinase (Figures 3A and 3B). This approach was designed to produce a functionally null allele, amenable to conditional rescue by expression of the corresponding cell-specific Cre. Immunochemical analysis of homozygous *Rgr*^S^ animals confirmed the absence of RGR in both the retina and the RPE (Figure 3C). *In vivo* assessment of retinal morphology by scanning laser ophthalmoscopy (SLO) and optical coherence tomography (OCT) identified no apparent ocular abnormalities in the animals up to 12 months of age (Figures 3D and 3E). Accordingly, histological examination of retinal sections from *Rgr*^+/+^ and *Rgr*^S/S^ animals, at ages from 1 to 12 months, revealed normal retinal morphology and comparable thickness of all retinal layers including the outer nuclear layer (ONL), indicating the absence of any detectable retinal degeneration associated with the RGR loss (Figures 3F and 3G). Since RGR provides a direct path for fast chromophore recycling, its loss could, however, affect retinoid flux under photic conditions. Excessive accumulation of all-*trans*-retinal is understood to have cytotoxic effects on photoreceptor cells.^24,25^

**Figure 4.**
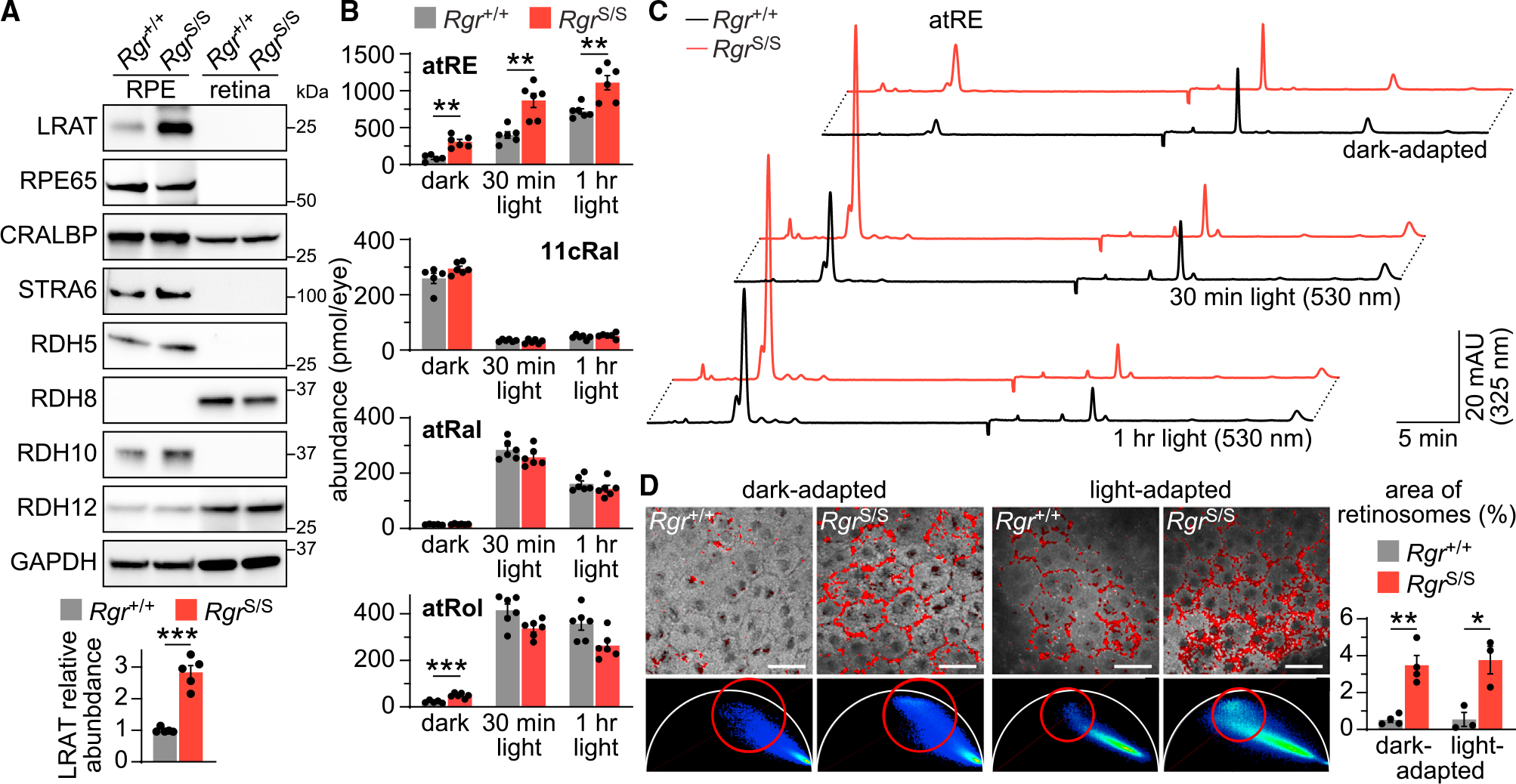
Absence of Rgr affects retinoid metabolism (A) Immunoblots of LRAT, RPE65, CRALBP, STRA6, RDH5, RDH8, RDH10, and RDH12 in retinal and RPE-eyecup extracts from 2-month-old *Rgr*^+/+^ and *Rgr*^S/S^ animals. The bar graph below shows quantification of LRAT in *Rgr*^+/+^ and *Rgr*^S/S^ RPEs. Values are plotted as mean ± SEM; ***p < 0.001 by Student’s t test; n = 5 independent experiments. (B) All-*trans*-retinyl ester (atRE), 11-*cis*-retinal (11cRal), all-*trans*-retinal (atRal), and all-*trans*-retinol (atRol) profiling of 2-month-old *Rgr*^+/+^ and *Rgr*^S/S^ eyes: dark adapted or after 30 min or 1 h of 530-nm light exposure. Values are plotted as mean ± SEM; n = 5 mice (dark), n = 6 mice (30 min and 1 h light); ***p < 0.001, **p < 0.01 by Student’s t tests with Holm-Šídák correction. (C) Representative chromatograms recorded at 325-nm light showing differential accumulation of atRE between the *Rgr*^+/+^ and *Rgr*^S/S^ eyes. (D) Distribution of retinoids in the RPE of 2-month-old *Rgr*^+/+^ and *Rgr*^S/S^ mice. Two-photon-excited (740-nm) fluorescence intensity images and respective FLIM phasor plots (underneath) obtained for dark- and daylight-adapted eyes. Red circles in each phasor plot indicate location of the retinosome phasor points, and corresponding image pixels were pseudo-colored (red) in the intensity images. Scale bars: 25 μm. The bar graph shows quantification of the areas occupied by retinosomes in *Rgr*^+/+^ and *Rgr*^S/S^ RPEs. Values are plotted as mean ± SEM; **p < 0.01, *p < 0.05 by Student’s t tests with Holm-Šídák correction; n = 4 (light), n = 3 (dark) independent experiments.

To assess how loss of RGR influences retinoid homeostasis, we first examined the status of key proteins involved in retinoid metabolism in the RPE and retina by immunoblotting. In agreement with previous studies of *Rgr* knockout mice,^26^ we observed significantly elevated levels of lecithin retinol acyltransferase (LRAT) in the RPE of *Rgr*^S/S^ animals. Levels of other classical visual cycle components were unaffected by the RGR loss (Figure 4A). To further evaluate the consequences of RGR deficiency for visual pigment regeneration, we conducted high-performance LC (HPLC) analysis of retinoids from *Rgr*^+/+^ and *Rgr*^S/S^ eyes after dark adaptation overnight or after prolonged exposure (30 min or 1 h) to 530-nm green light. Both dark-adapted and light-exposed eyes that lacked RGR showed unchanged all-*trans*-retinal levels but did show significant all-*trans* retinyl ester accumulation (Figures 4B and 4C). This observation can be attributed to elevated LRAT levels in the RPE of *Rgr*^S/S^ animals. Thus, LRAT upregulation, adapting to RGR absence, could constitute a protective mechanism by which the retina compensates for its capacity to process increased amounts of chromophore released from photoreceptors during light exposure to prevent all-*trans*-retinal cytotoxicity to the retina. In line with this observation, scRNA-seq analysis of a dataset generated from our previous study^27^ revealed that light-sensitive *Abca4*^−/−^*Rdh8*^−/−^ mice demonstrate upregulation of RGR, in both RPE and in Müller glia, following exposure to bright light (Figure S3B).

To further explore the relationship between RGR and retinyl ester biology, freshly dissected RPE flatmounts were subjected to two-photon-excited fluorescence imaging.^28^ Using 740-nm two-photon excitation and fluorescence lifetime imaging (FLIM), we analyzed the RPE in eyes from both *Rgr*^+/+^ and *Rgr*^S/S^ mice, either dark or light adapted. This approach revealed the presence of previously characterized phasor signatures of retinyl esters located inside the universal semicircle, as well as the absence of di-retinal conjugate A2E signatures^29^ (Figure 4D). Mapping of the phasor signature signals on the fluorescence intensity images from all samples yielded patterns consistent with the distribution of retinosomes, which are sites of highly concentrated retinyl ester storage.^30^ In all cases, the area occupied by retinosomes was significantly larger in the *Rgr*^S/S^ animals under both dark- and light-adapted conditions (Figure 4D). This result is in agreement with the larger content of retinosomes resulting from Rpe65 knockout and with the lack of retinosomes in *Lrat* knockout mice.^30^ Taken together, these observations suggest that besides the chromophore-regeneration aspect of RGR activity, its ability to act as a very efficient sink for all-*trans*-retinal under sustained light conditions ensures the health and proper function of photoreceptor cells. The loss of RGR likely activates a compensatory mechanism in the mouse retina involving the upregulation of an alternative all-*trans*-retinoid utilization pathway through LRAT, leading to elevated retinyl ester storage in the RPE.

### *Rgr*^S^ allele facilitates controlled rescue of RGR in cell types of interest

**Figure 5.**
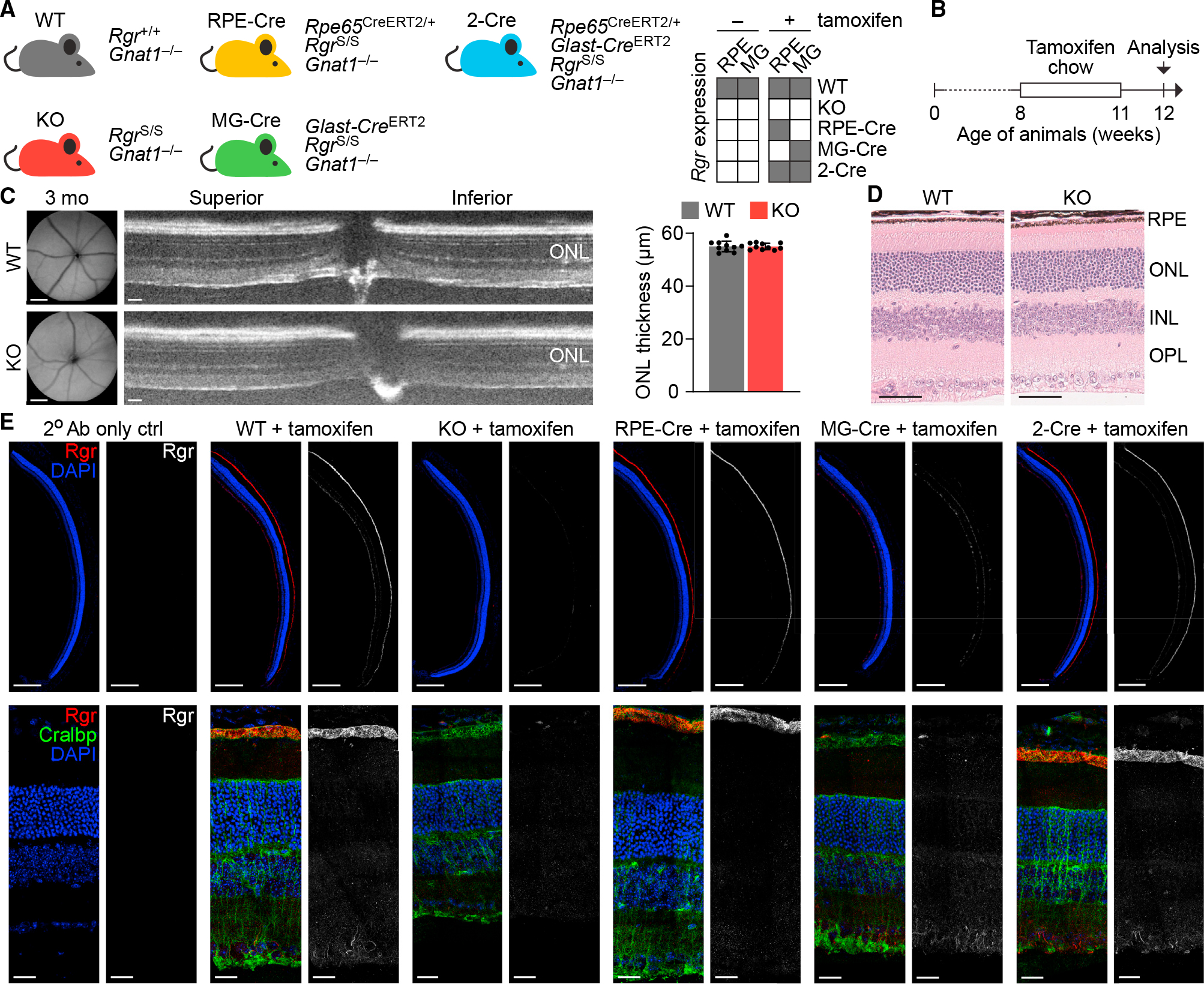
Conditional gene rescue restores *Rgr* expression in the RPE and in Müller glia (A) Genotypes of mouse lines developed to characterize the contribution of *Rgr* to cone visual function. Lines carrying *Rpe65* and *Glast* promoter-driven tamoxifen-inducible Cre recombinase transgenes (CreERT2) were used to selectively restore expression of *Rgr* in the RPE or in Müller glia (MGs), respectively. Schematic heatmap (right) shows anticipated status of *Rgr* expression before and after the tamoxifen administration. (B) Experimental schedule for the tamoxifen treatment to induce Cre-mediated conditional rescue of *Rgr*. (C) SLO (left, scale bars: 500 μm) and retinal OCT (middle, scale bars: 50 μm) images of WT and KO eyes, at the time of structural and functional analysis (3 months), showing no features that distinguish the two lines carrying the *Gnat1*^−/−^ background; OCT-based quantification of ONL thickness (panel at the right, data are shown as mean ± SEM, n = 10 eyes). (D) H&E-stained retinal sections of 3-month-old WT and KO animals, showing no signs of retinal structural pathology. Images were taken approx. 500 μm from the ONH. Scale bars: 50 μm. (E) IHC images of eye cryosections from WT, KO, RPE-Cre, MG-Cre, and 2-Cre animals (genotype details indicated in A) after tamoxifen treatment, stained tovisualize RGR, CRALBP, and nuclei (DAPI). For each mouse/treatment combination, the left column shows images combined for all 3 targets; the right column shows only RGR distribution. RGR, absent in the KO mouse line, is selectively restored in the RPEs of RPE-Cre mice, the MGs of MG-Cre mice, and both cell types of the 2-Cre mice. Low-magnification images (top row, scale bars: 100 μm) are presented in central (ONH, top)-to-peripheral (bottom) orientation and were cropped to visualize only retina and RPE. High-magnification images (bottom row, scale bars: 20 μm) were taken 250–500 μm from the ONH.

Previous studies on global *Rgr* knockout animals demonstrated the crucial role of RGR in retaining cone photoreceptor sensitivity in daylight conditions.^9,15,16^ Considering the existence of separate pools of RGR in RPE cells and Müller glia (Figure 2B), we sought to distinguish their relative contributions to cone function. We first bred the *Rgr*^S/S^ mice onto the rod G-protein transducin α-subunit knockout (*Gnat1*^−/−^) background to dissect the cone-driven response in the retina.^31^ These animals were further crossed with two transgenic lines, *Rpe65*^CreERT2^ and *Glast-Cre*^ERT2^, to selectively induce Cre-dependent recovery for RGR expression in the RPE and in Müller glia, respectively.^32,33^ In total, we generated five mouse lines (Figure 5A), all carrying the Gnat1^/^ background, referred to as wild type (WT; *Rgr*^+/+^), knockout (KO; *Rgr*^S/S^), RPE-Cre (*Rgr*^S/S^, *Rpe65*^CreERT2/+^), MG-Cre (^RgrS/S^, *Glast-Cre*^ERT2^), and 2-Cre (*Rgr*^S/S^, *Rpe65*^CreERT2/+^, *Glast-Cre*^ERT2^). While the KO line remained an effective *Rgr* KO, irrespective of the experimental condition, the three Cre-driver lines supported conditional *Rgr*-gene rescue, specifically in the RPE (RPE-Cre), in the Müller glia (MG-Cre), and in both cell types (2-Cre), upon tamoxifen treatment (Figure 5B). Retinal morphology of WT and KO animals assessed by SLO, OCT (Figure 5C), and histology (Figure 5D) at 3 months of age showed no apparent retinal abnormalities arising from the dual KO of both *Gnat1* and *Rgr*.

RNA *in situ* hybridization (ISH) on retinal sections from WT animals revealed robust expression of *Rgr* in RPE cells (Figure S4), in line with the immunoblotting data (Figure 3C). *Rgr*-mRNA signal originating from the inner nuclear layer (INL), where the Müller glia nuclei reside, appeared much weaker than in the RPE and scarcer compared to the *Rlbp1* counterstain. As expected, *Rgr* expression was lost in the KO eyes but mimicked WT levels in RPE cells of the RPE-Cre line, Müller glia of the MG-Cre line, and both RPE and Müller glia of the 2-Cre line after tamoxifen treatment (Figure S4). To confirm these observations at the protein level, we performed immunohistochemical (IHC) staining on analogous retinal sections (Figure 5E). While we observed an even distribution of RGR in RPE cells of WT eyes, its presence in the Müller glia was detectable only in a fraction of cells, concentrated primarily in the endfeet and thin stalks extending toward the INL. Both ISH and IHC data confirmed the existence of a subpopulation of Müller glia with RGR expression, as well as the successful recovery of RGR in all three Cre driver lines (RPE-Cre, MG-Cre line, 2-Cre) upon tamoxifen induction.

### Photic visual pigment regeneration supports cone function in bright light and accelerates subsequent dark adaptation

**Figure 6.**
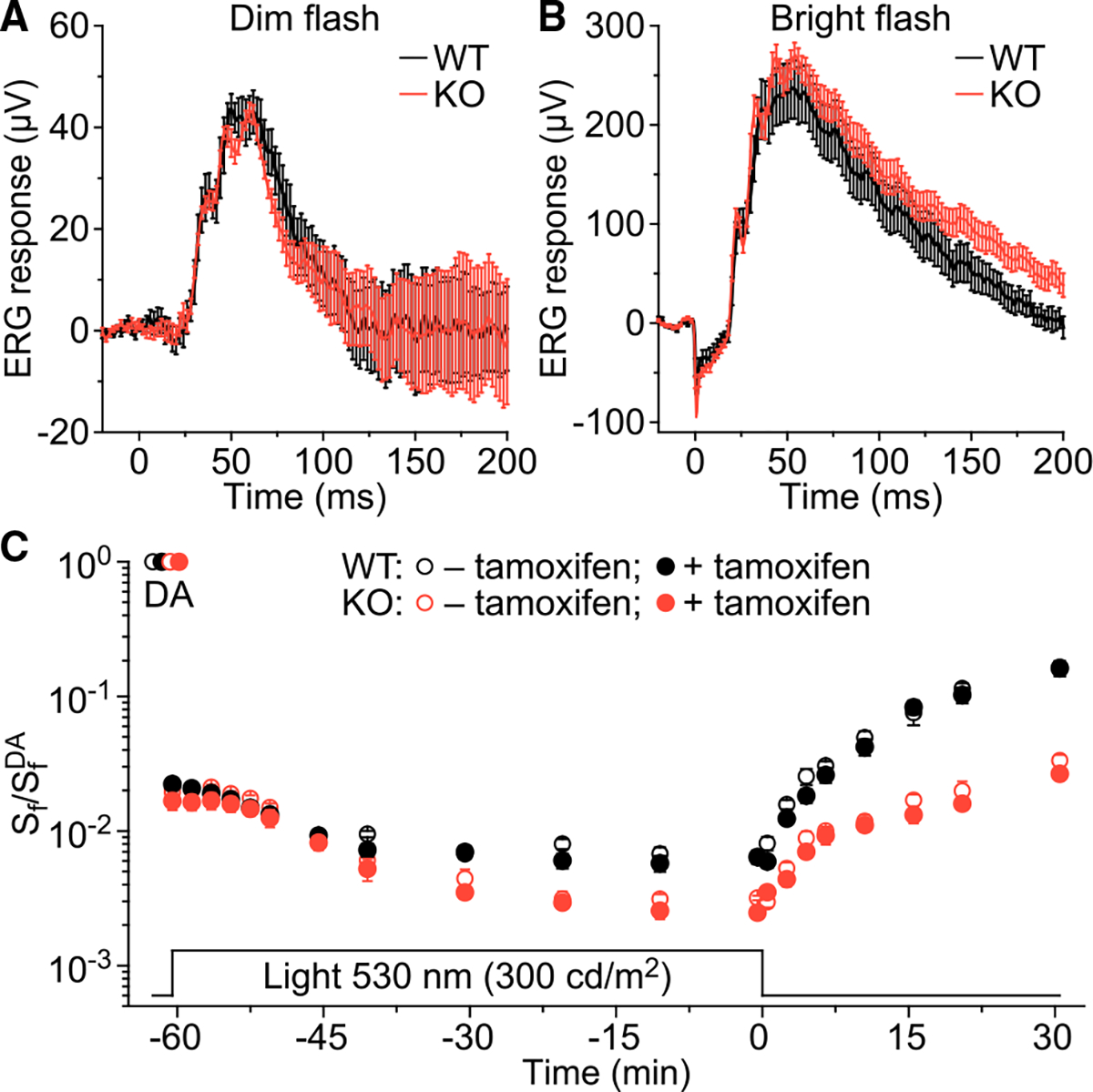
Loss of RGR compromises the sustained function of cones in steady background light and their dark adaptation *in vivo* (A) Population-averaged (mean ± SEM) dim-flash M-cone-driven ERG b-wave responses to test stimuli of 0.24 cd s m^−2^ for dark-adapted WT (n = 10 eyes) and KO (n = 12 eyes) mice. WT and KO genotype details are indicated in Figure 5A. (B) Comparison of population-averaged (mean ± SEM) M-cone-driven ERG b-wave responses to a bright flash (700 cd s m^−2^) for WT and KO animals the same as in (A). (C) Cone-driven ERG b-wave flash sensitivity (*S*_f_) following illumination with green 530-nm background light (300 cd m^−2^, 60 min), and its subsequent recovery in the dark in WT and KO mice either treated (+) or not treated (−) with tamoxifen. WT and KO genotype details are indicated in Figure 5A; WT −tamoxifen: n = 10 eyes, WT +tamoxifen: n = 14 eyes, KO −tamoxifen: n = 12 eyes, KO +tamoxifen: n = 14 eyes. *S*_f_ was normalized to the corresponding dark-adapted value (*S*_f_^DA^) in each case. The time course of light exposure is shown on the bottom. Data are expressed as mean ± SEM (error bars are often smaller than symbol size); significant differences were associated with time point (p < 0.0001) and genotype (p < 0.0001) but not with tamoxifen treatment; three-way repeated measures ANOVA (Table S1).

To begin evaluating the role of RGR in cone photoreceptor function, we first performed *in vivo* electroretinography (ERG) recordings with WT and KO mice. The *Gnat1*^−/−^ background in those animals ablated the rod component and enabled study exclusively of the cone component of the ERG response. Recordings from dark-adapted WT and KO mice revealed that the amplitude and waveform of both dim-flash (Figure 6A) and bright-flash (Figure 6B) M-cone-driven ERG b-wave responses were not affected by the loss of RGR. This result was expected, as the expression level of most of the retinoid metabolism proteins remains unaffected in these animals (Figure 4A). Next, we monitored the flash sensitivity of cone-driven b-wave in animals exposed to 300-cd/m^2^ background 530-nm light for 60 min. This protocol was designed to continuously bleach a fraction of the cone visual pigment while simultaneously activating RGR. We found that in WT animals, the onset of the background light resulted in an initial ~50-fold decrease in M-cone b-wave sensitivity due to initial rapid depletion of the cone pigment by light, followed by a gradual decline in cone function until it reached a plateau within 30 min of the background light exposure (Figure 6C). We observed a similar initial sensitivity decline in KO cone responses (Figure 6C). However, the subsequent gradual desensitization in steady background light was more pronounced, reaching a plateau in sensitivity about half that of WT. Tamoxifen treatment did not affect the level of cone desensitization in both WT and KO mice (Figure 6C; Table S1), ruling out any drug-related effects in our experiments. Finally, to evaluate the effect of RGR on subsequent dark adaptation, the background light was turned off after 60 min (Figure 6C, 0 min time on the plot), and the recovery of M-cone b-wave sensitivity was tracked for an additional 30 min while the cones were dark adapting. We observed that, regardless of tamoxifen treatment, cone dark adaptation in KO mice was substantially suppressed compared with that in WT mice, with an ~5 times lower level of cone sensitivity by 30 min. Together, these results indicate that RGR’s role in the recycling of chromophore in continuous bright background light has an extended effect onto subsequent dark adaptation of cone photoreceptors.

### Sustained cone function in bright light depends on both RPE and Müller glia RGR pools

**Figure 7.**
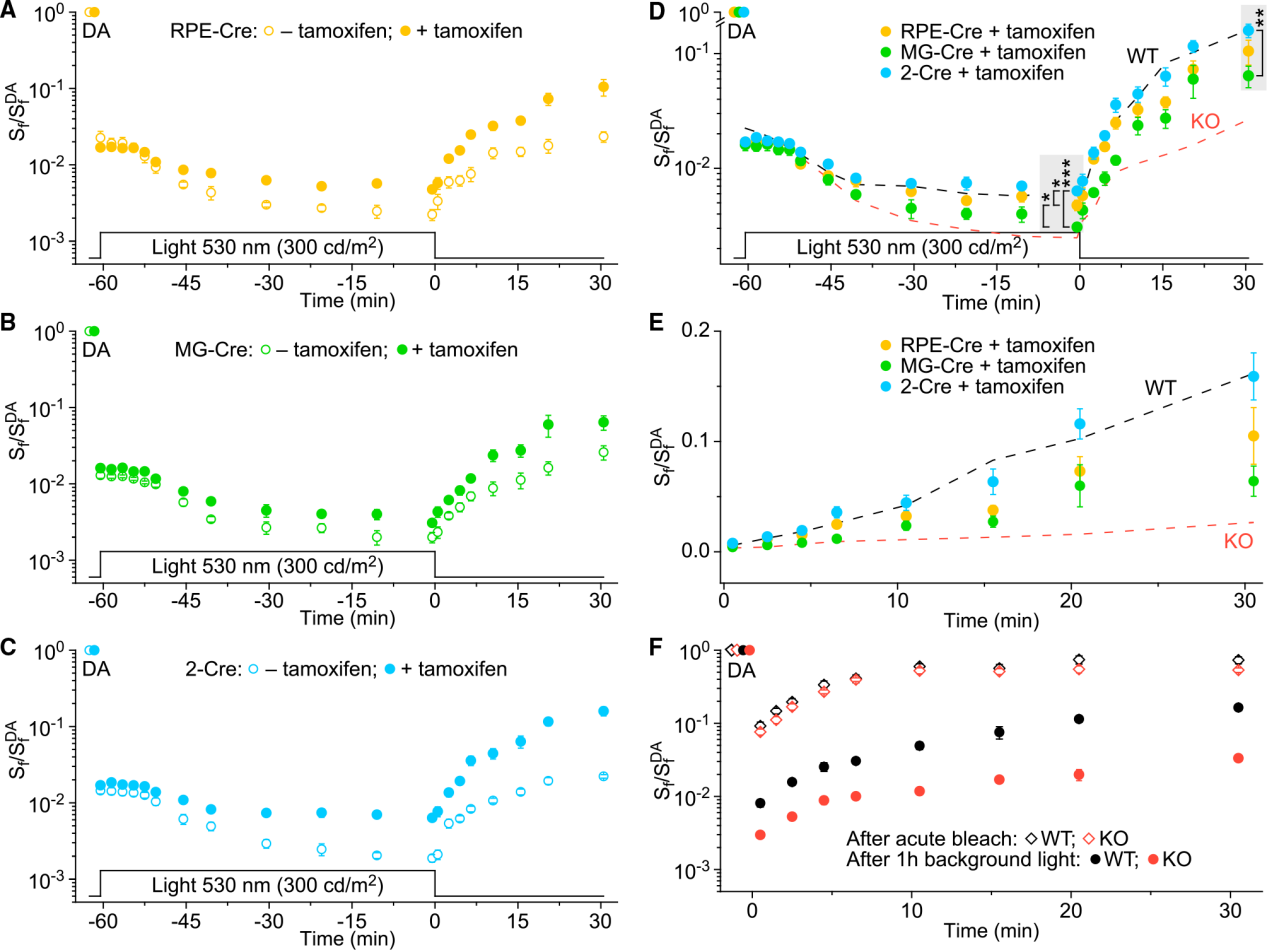
Rescue of RGR in RPE and/or Müller cells restores the sustained function of cones in background light and their dark adaptation *in vivo* (A–C) Changes in M-cone-driven ERG b-wave *S*_f_ *in vivo* following illumination with green 530-nm background light (300 cd m^−2^, 60 min), and its subsequent recovery in the dark in RPE-Cre (A), MG-Cre (B), and 2-Cre (C) animals, either treated (+) or not treated (−) with tamoxifen. Genotype details are indicated in Figure 5A; respective numbers of mouse eyes tested (n) are indicated in Table S1. *S*_f_ was normalized to the corresponding *S*_f_^DA^ in each case. The time course of light exposure is shown on the bottom. Data are presented as mean ± SEM (error bars are often smaller than symbol size); significant differences were associated with two factors: time point and mouse group; two-way repeated measures ANOVA (Table S1). (D) Comparison of relative rescue of cone function in steady background light and subsequent dark adaptation upon recovery of RGR expression in the RPEs and/or MGs. Responses for tamoxifen-treated RPE-Cre (n = 16 eyes), MG-Cre (n = 12 eyes), and 2-Cre (n = 12 eyes) mice are replotted from (A), (B), and (C), respectively. Dashed lines represent averaged data for WT and KO animals replotted from Figure 6C. All genotype details are indicated in Figure 5A. Significant differences were associated with two factors: time point (p < 0.0001) and mouse group (p < 0.01); two-way repeated measures ANOVA (Table S1). For time points indicated with gray shaded rectangles, representing the end of the background light illumination phase (−0.5 min) and the end of the dark adaptation phase (30.5 min), significant differences are indicated as follows: ***p < 0.001, **p < 0.01, *p < 0.05, Holm-Šídák post hoc test. (E) Dark adaptation portion of the data shown in (D), presented on an extended timescale and a linear *S*_f_ scale. (F) Comparison of M-cone dark adaptation in untreated WT (n = 10 eyes) and KO (n = 12 eyes) mice *in vivo* after extended exposure to bright light (closed symbols, replotted from Figure 6C) and after acute bleaching of >90% of the cone pigment at time 0 with 530-nm light (open symbols, n = 10 for both WT and KO). WT and KO genotype details are indicated in Figure 5A. *S*_f_^DA^ designates the corresponding dark-adapted sensitivity in each case. Data are presented as mean ± SEM (error bars are often smaller than symbol size).

Next, we sought to determine the relative contribution of RGR in the RPE and Müller cells to the regeneration of visual pigment in cones by selectively restoring RGR expression in each of the two cell types. Control RPE-Cre animals that were not treated with tamoxifen showed M-cone function comparable with that of KO animals (Figure 7A). Upon tamoxifen-induced RGR rescue in the RPE cells, RPE-Cre mice showed significant improvement, both in the level of steady-state desensitization during background light exposure and in the extent of the subsequent recovery of cone sensitivity in darkness (Figure 7A; Table S1). This result shows that the RGR isomerase activity originating from the RPE contributes to the supply of active chromophore to cones. Similarly, we observed significantly improved cone function, in sustained light and in dark adaptation, of MG-Cre mice treated with tamoxifen (Figure 7B; Table S1). This result demonstrates that RGR in the Müller glia likewise contributes to the supply of active chromophore to cones. Finally, we investigated the function of cones in 2-Cre mice where RGR expression was restored both in the RPE and in Müller cells. The experiment resulted in robust improvement of cone function, in steady background and in dark adaptation in tamoxifen-treated mice, to a level comparable to that of the WT animals (Figure 7C; Table S1). As expected, with lack of tamoxifen treatment, all three Cre-driver lines exhibited responses comparable with KO animals (Figure S5). Together, these results show that both RPE and Müller glial RGR pools are essential for the efficient supply of active chromophore to cone photoreceptors.

To evaluate the relative contribution of the two RGR pools to the function of cones, we directly compared the cone desensitization levels in all five mouse lines. Unsurprisingly, KO mice were desensitized most severely, while an increasing improvement in cone function was observed upon selective restoration of RGR expression in Müller glia, the RPE, and both cell types (Figure 7D). Within the second half of the background light-exposure period (Figure 7D, experimental points from −30.5 to −0.5 min), when cone responses reached an apparent plateau, their level was on average ~1.4-fold higher in MG-Cre animals, ~1.9-fold higher in RPE-Cre animals, and ~2.5-fold higher in the 2-Cre mouse line compared with in KO animals. After steady background illumination for 60 min, cone function remained significantly attenuated in both MG-Cre and RPE-Cre animals compared with the 2-Cre line, suggesting that RPE and Müller glia RGR pools play non-redundant roles in the 11-*cis*-retinoid supply to cones. At the same time, the observed cone sensitivity levels were significantly higher in the RPE-Cre mice than in the MG-Cre animals. The subsequent recovery of cone sensitivity in darkness followed the same pattern, occurring the slowest in the KO animals and progressively increasing in MG-Cre, RPE-Cre, and 2-Cre mice (Figures 7D and 7E, experimental points from 0.5 to 30 min). By the end of the dark adaptation period in this experiment, only MG-Cre animals exhibited significantly attenuated cone responses compared with the 2-Cre line. Overall, the results demonstrate that both the RPE and Müller glia are involved in the photic visual cycle in the mammalian retina. In mice, the RGR contribution from the RPE exceeds that of the Müller cells, which likely reflects the differences in relative RGR abundance between the two cell types. Moreover, loss of RGR does not cause any long-term deleterious effects on cone function, which can be fully restored with replenishment of RGR in the Müller cells and in the RPE.

Although the mice were not exposed to light that could stimulate RGR during the dark adaptation period, cone recovery was suppressed by the absence of RGR in the RPE and/or in Müller glia. To verify directly whether RGR modulates the recovery of cone sensitivity in darkness, we evaluated the dark adaptation of M-cones following an acute bleach by exposure to bright 520-nm light for 35 s. We found that the cone recovery in this case was significantly faster than the recovery following 60 min of background light, and it was not affected by the absence of RGR (Figure 7F). We conclude that *in vivo* RGR modulates the chromophore supply in prolonged background light, which can also modulate the subsequent cone dark adaptation; however, RGR does not affect cone pigment regeneration following a brief exposure to bright light.

### Absence of RGR suppresses dark adaptation of rods after extended illumination

**Figure 8.**
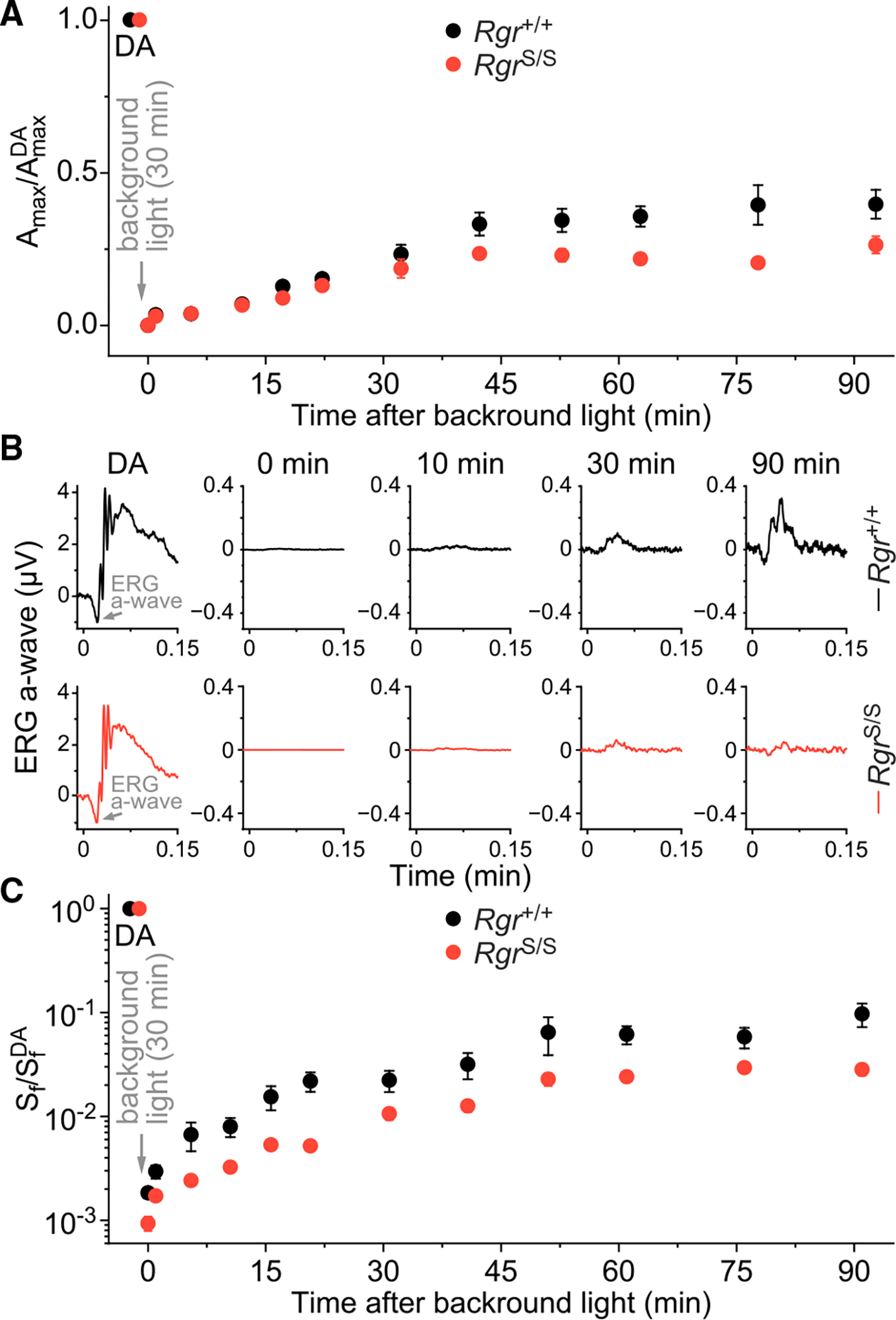
Absence of RGR suppresses rod dark adaptation *in vivo* (A) Recovery of normalized scotopic ERG maximal a-wave amplitudes (*A*_max_) in the dark, following illumination with green 530-nm background light (300 cd m^−2^, 30 min) bleaching >90% of rhodopsin, in control *Rgr*^+/+^ (*Gnat1*^+/+^, n = 12 eyes) and embryonic RGR KO *Rgr*^S/S^ (*Gnat1*^+/+^, n = 10 eyes) mice. *A*_max_^DA^ designates the maximal response of dark-adapted rods. (B) Representative rod dim-flash responses in the dark and at four time points after illumination with the same bleaching light as described in (A) in *Rgr*^+/+^ (*Gnat1*^+/+^, top) and *Rgr*^S/S^ (*Gnat1*^+/+^, bottom) mice. For each time point, responses were divided by corresponding test flash intensities (in cd s m^−2^) and their prebleach dark-adapted maximal ERG a-wave amplitudes (in μV), followed by normalization to respective fractional *S*_f_ in darkness. (C) Recovery of normalized scotopic ERG a-wave *S*_f_ in darkness, following green light illumination, in *Rgr*^+/+^ (*Gnat1*^+/+^, n = 12 eyes) and *Rgr*^S/S^ (*Gnat1*^+/+^, n = 10 eyes) mice. Animals and experimental conditions were the same as in (A) and (B). *S*_f_^DA^ designates the sensitivity of dark-adapted rods. Data in (A) and (C) are presented as mean ± SEM (error bars are often smaller than symbol size). Significant differences were associated with two factors: time point and genotype; two-way repeated measures ANOVA (Table S1).

Finally, we evaluated whether RGR plays a role in the regeneration of visual pigment in rods. For this purpose, we exposed *Rgr*^+/+^ and *Rgr*^S/S^ mice (carrying the *Gnat1*^+/+^ background) to bright light to trigger bleaching of their visual pigment and RGR-driven chromophore photoproduction. Given rod response saturation under these conditions, we were only able to measure the subsequent recovery at the end of the background exposure. We monitored the restoration of the rod-driven ERG a-wave in animals after exposure to 300-cd/m^2^ 530-nm background light for 30 min. The recovery of the maximal response of the scotopic a-wave was suppressed in *Rgr*^S/S^ animals relative to *Rgr*^+/+^ controls (Figure 8A). Similarly, dim flash responses periodically obtained during the dark recovery were consistently smaller in RGR-deficient mice compared with in controls (Figure 8B). Rod a-wave flash sensitivity measurements obtained from these responses also demonstrated suppressed dark adaptation in RGR-deficient animals compared with the WT (Figure 8C). Since the RPE65 levels were not affected by RGR loss in our animal model (Figure 4A), these results show that the RGR pool in the RPE is important for the efficient supply of chromophore to rod photoreceptors and that it accelerates their dark adaptation upon the transition from bright light to darkness.

## DISCUSSION

Despite the similarity in pigment concentration, density, and bleaching levels upon bright light exposure, cones restore their sensitivity about 10 times faster than rods.^5^ The RPE-based classical visual cycle, the best-characterized pathway of *trans-cis* retinoid isomerization, is too slow to meet the chromophore-regeneration demand of the cones under sustained light conditions.^4^ The RGR-dependent photic visual cycle is the only prominent alternative source of *trans-cis* isomerase activity in the retina known to date. Initial characterization showed that purified RGR generates 11-*cis*-retinal at a relatively slow rate, which decreases further after the first 30 s of light exposure.^10^ However, a more recent study estimated that photic RGR activity in a native membrane environment, in the presence of CRALBP, far exceeds the rate of 11-*cis*-retinol formation by the RPE in the dark.^9^ RGR has been understood to be a bistable opsin^34^; however, the extent to which RGR behaves as a bleaching opsin to release of 11-*cis*-retinal and support visual pigment recycling has not been thoroughly characterized. Here, we demonstrate the bleaching properties of RGR, measuring the rate of hydrolysis of the RGR* to quickly generate 11-*cis*-retinal. This hydrolysis occurred approximately 60-fold faster than the hydrolytic release of all-*trans*-retinal from rod opsin, the rate-limiting step of visual pigment bleaching,^19^ suggesting that the photic visual cycle driven by RGR is sufficient to fill the gap between the RPE65-mediated chromophore supply rate and the demand generated by cone photoreceptors under sustained light conditions.

The high affinity for and rapid photic processing of all-*trans*-retinal predispose RGR to act as an effective scavenger for bleached chromophore, whose levels increase rapidly upon bright light exposure.^35,36^ Notably, elimination of RGR led to a significant overexpression of LRAT, an alternative all-*trans*-retinoid utilization pathway. In humans, LRAT transcription has been shown to significantly increase in response to all-*trans*-retinal accumulation.^37^ This further suggests that RGR plays a critical role in sequestering and processing all-*trans*-retinal in photic conditions, thereby mitigating the cytotoxic potential related to its accumulation. It is known that at least a portion of all-*trans*-retinal released from opsins undergoes reduction to retinol within the photoreceptor outer and inner segments,^38^ relatively faster in cones than in rods.^39,40^ Thus, in terms of substrate availability for RGR, the direct utilization of bleached chromophore could be complemented by oxidation of all-*trans*-retinol in RPE and Müller cells. While RGR and CRALBP are coexpressed in both the RPE and in Müller glia, these two cell types differ significantly in their expression of other retinoid metabolism proteins, including RDH enzymes. Consequently, the photic visual pigment-regeneration pathway can involve different sets of auxiliary members, depending on in which cell type it occurs. For instance, RGR has been shown to interact with RDH10 expressed in both cell types,^16^ as well as RDH5 expressed exclusively in the RPE.^41^ The observation that only a subset of Müller glia express RGR in macaque retina provided us with a unique opportunity to investigate how this subpopulation adjusts to support the activity of RGR. The RGR+ cells share a unique transcriptional profile, involving upregulation of RDH11 and CRALBP among other transcripts. RDH11 *in vitro* demonstrates equal specificity toward both *cis*- and all-*trans*-retinoids^23^ but is more catalytically efficient in the reductive, rather than the oxidative, direction in the presence of the appropriate cofactor.^42^ Nevertheless, its function *in vivo* is likely further dictated by the actual concentrations of substrates and by cofactors supporting either oxidation or reduction: NADP^+^ and NADPH, respectively. While typically these cofactors exist over-whelmingly in the reduced form in the cytosol, light exposure of cultured Müller glia has been shown to significantly increase the intracellular NADP^+^/NADPH ratio.^43^ As such, the source of all-*trans*-retinal for RGR could derive from RDH-mediated oxidation of all-*trans*-retinol produced from bleaching of photoreceptors.^9^ Notably, RDH11 is the most abundant RDH in human Müller glia, and its upregulation in the RGR+ subpopulation suggests that RDH11 could play an important role in supplying the substrate for RGR to facilitate photic visual pigment regeneration. Likewise, the upregulation of CRALBP likely reflects its importance in preserving the 11-*cis*-retinal from reisomerization.

Cones, but not rods, can regenerate their visual pigment independently of the RPE, and the additional retinoid isomerization capability originates in the Müller glia.^44,45^ This observation, recognized as cone-specific intraretinal visual cycle^5^ and initially attributed to the activity of dihydroceramide desaturase DES1,^46^ can now be explained instead by the photic visual pigment regeneration mediated by RGR. In this study, we demonstrated that the RGR *trans-cis* isomerase activity originates from both Müller glia and RPE cells, influencing the function of both cones and rods. Whether any intercellular communication occurs between the Müller and RPE cells to coordinate their involvement in photic visual pigment regeneration remains unclear; however, the RGR pools in these respective cell types did not exhibit redundancy in supporting sustained cone sensitivity. During prolonged light exposure and subsequent dark adaptation, RGR expressed in mouse RPE supported cone sensitivity to a greater extent than the RGR expressed in Müller glia. While this finding could, in part, reflect the higher amount of RGR present in the mouse RPE relative to the Müller cells, the differences observed at the protein level appear to greatly exceed the differences in physiological responses. One potential explanation is that the RGR contribution could be affected by differing amounts of substrate available in the two cell types. RGR is uniformly distributed across the RPE; however, in Müller glia, it is concentrated in a subpopulation of cells. Thus, it is conceivable that this expression pattern in Müller glia, sparse in some species such as mouse and macaque, is not random; rather, it could be regionally associated with the localization of cones in the retina, thereby enabling privileged access to *trans-cis* isomerization under photic conditions. In line with this hypothesis human RGR+ Müller glia exhibit higher expression of RGR in the cone-rich foveal than peripheral region.^22,47^ Alternatively, differences in the capability to oxidize all-*trans*-retinol by the two cell types could affect the extent to which each of the two RGR pools contributes to chromophore regeneration. Finally, as RGR in the RPE supports both rod- and cone-pigment regeneration in bright light, competition for the RGR-derived chromophore between the two photoreceptor types could limit the contribution from the RPE pool of RGR to pigment regeneration in cones.

As is becoming increasingly clear, the classical and photic visual cycles, to some degree, support continuous chromophore supply to both rods and cones.^48^ While rod function critically depends on the chromophore regeneration through the RPE65-mediated pathway, its inhibition also suppresses the later phase of cone dark adaptation.^49^ The photic cycle contributes significantly to cone function under sustained light yet, in addition, enables faster dark adaptation of both rods and cones upon a sharp switch to dimmer light environment. Finally, the possibility that Müller glia could be classified into distinct subtypes, based in part on the RGR expression status, opens avenues for the study of Müller glia heterogeneity regarding their numerous other functions in the retina.

### Limitations of the study

The study was primarily focused on the contribution of RGR to cone function, as photic conditions directly support the photoisomerase activity of RGR. While we were able to show that loss of RGR affects the speed of dark adaptation of the rods, we did not establish the relative contribution to this process of the separate RGR pools in Müller glia and the RPE. Comparison of the RGR+ and RGR− Müller glia single-cell transcriptomes was performed only on the data from macaques; therefore, it is possible that other DEGs would be associated with the RGR expression status in Müller glia of other species. In mouse eyes, the Müller glia constitute a much smaller fraction of the retina than in human eyes, so their efficient sequencing will require a more targeted approach.

## STAR★METHODS

### RESOURCE AVAILABILITY

#### Lead contact

Further information and requests for resources and reagents should be directed to and will be fulfilled by the lead contact, Krzysztof Palczewski (kpalczew@uci.edu).

#### Materials availability

The *Rgr*^Stop^ (C57BL/6-Rgr^tm1.1Kpal/J^) mouse line generated in this study has been deposited with the The Jackson Laboratory (Stock # 038172).

#### Data and code availability

- Mouse single-cell RNA-seq data generated in this study have been deposited at Gene Expression Omnibus (GEO) and are publicly available as of the date of publication. Accession numbers are listed in the key resources table. Other data reported in this paper will be shared by the lead contact upon request.
- All original code has been deposited at Github repository and is publicly available as of the date of publication. DOIs are listed in the key resources table.
- Any additional information required to reanalyze the data reported in this work paper is available from the lead contact upon request.

### EXPERIMENTAL MODEL AND STUDY PARTICIPANT DETAILS

#### Experimental animals

All animal procedures complied with the NIH Guide for the Care and Use of Laboratory Animals and the ARVO Statement for the Use of Animals in Ophthalmic and Vision Research; and were approved by the Institutional Animal Care and Use Committee of UC Irvine (protocols AUP-21–096 and AUP-21–031). The following previously described mouse strains were used in this study: C57BL/6J (Jackson Laboratory), *Gnat1*^−/−^ carrying the L450 *Rpe65* isoform,^31^ *Rpe65*^CreERT2^ (C57BL/6-*Rpe65*^tm1.1(cre/ERT2)Kser^/J, Jackson Laboratory),^32^ and *Glast-Cre*^ERT2^ (Tg(Slc1a3-cre/ERT)1Nat/J, Jackson Laboratory).^33^ Animals were housed under 12-h/12-h light/dark cycles and fed a standard soy protein-free diet (Teklad 2020X, Envigo) *ad libitum*. All *in vivo* and *in vitro* experiments were performed on both male and female adult mice. All animals were drug- and test-naïve.

#### *Rgr*^S^ mouse

The *Rgr*^S^ allele was generated by homologous recombination of a Stop cassette flanked by two *loxP* sites into intron 1 of *Rgr* in iTL IN2 (C57BL/6) embryonic stem cells (Ingenious Targeting Laboratory). A neomycin-resistance gene integrated with the Stop cassette was used to select targeted cells, which were further microinjected into Balb/c blastocysts. Resulting chimeras with a high percentage of black coat color were backcrossed onto the C57BL/6J background. The integrity of the targeted region was verified by amplifying (PCR) and sequencing (Sanger) the integration sites of DNA from ear samples, using primers listed in Figure 3A and Table S2. Primer pairs a/b and c/d enabled amplification of DNA proximal (0.67 kB) and distal (1.32 kB) to the Stop cassette integration sites, respectively. Colony founders were screened for known mutations in *Pde6b* (rd1) and *Crb1* (rd8), and all strains developed on the *Gnat1*^−/−^ background were backcrossed to carry the L450 Rpe65 variant. *Rgr*^S^ mouse was transferred to the Repository at The Jackson Laboratory (strain #: 038172).

### METHOD DETAILS

#### Proteinase K digest

The following method was adapted from Hong et al.^19^ to determine the chromophore location in RGR from bovine RPE microsomes, before and after light exposure. RPE cells were isolated from bovine eye cups by gentle brushing in cold buffered sucrose (0.25 M sucrose, 25 mM Tris-acetate, pH 7, 1 mM dithiothreitol), and the microsomal fraction was isolated from the cell homogenates by differential centrifugation: 20,000*g* for 20 min at 4°C followed by 150,000*g* spin for 1 h at 4°C.^57^ The resulting RPE-microsomal pellet was resuspended in 10 mM Bis-tris propane, pH 7.4 to achieve a total protein concentration of ~5 mg/mL. In the dark room under dim red light, RPE microsomes were incubated for 15 min with 20 μM all-*trans*-retinal (MilliporeSigma) to regenerate the RGR all-*trans*-retinylidene adduct. Then two aliquots were prepared with one kept in the dark and another exposed for 10 s to 530 nm light (approximate *λ*_max_ of the action spectrum of RGR^9^) from a fiber-coupled LED (Thorlabs) set to an intensity of 125 μW using a T-Cube LED driver (Thorlabs). Both mixtures were kept at 0°C to prevent any chromophore hydrolysis. Subsequently, 3 parts by volume of NaBH_4_ (Fisher Scientific) in *i*PrOH (Fisher Scientific) was added to 1 part of RPE microsomes for immediate reduction of the Schiff base to trap the chromophore as a non-hydrolyzable retinyl amine moiety of RGR while also isolating RGR by protein precipitation. The precipitated protein pellet was washed with methanol (MeOH, Fisher Scientific), followed by water. The protein pellet was then resuspended in proteinase K buffer (4 M urea, 100 mM bis-tris propane (BTP) pH 7.8, 100 mM CaCl_2_). Subsequently, proteinase K (Viagen Biotech) was added at approximately 10 times the weight of the RGR substrate. The digestion mixture was incubated for 1 h at 37°C, followed by 23 h at 20°C with gentle agitation using a thermomixer set at 750 rpm. The resulting digestion mixture was desalted using a BioPure SPN C18 spin column (The Nest Group). The column was washed with 20% acetonitrile (ACN) in water with 0.1% formic acid (FA), and peptides were eluted using 60% ACN. The N^ε^-retinyl-peptide products in the eluent were separated using a Vanquish Flex HPLC system (Thermo Fisher Scientific) with a XBridge C18 column (Waters) and a 40 min gradient of 20%–60% ACN in water with 0.1% FA at a flow rate of 0.3 mL/min. N^ε^-retinyl-peptide products were detected by HPLC absorbance at 330 nm and identified by MS/MS with CID fragmentation using the LTQ XL (Thermo Fisher Scientific). The isomeric identity of the retinyl moiety of the peptides was also verified by their absorbance spectrum, using the 1260 Infinity HPLC system (Agilent) with the same chromatographic conditions.

#### Pronase digest

The following method was adapted from Hong et al.^19^ to determine the isomeric composition of the retinylidene chromophore of RGR in bovine RPE microsomes, before and after exposure to light. Samples were prepared and processed as for proteinase K digest, with the following changes. After protein precipitation and washing with MeOH and water, the protein pellet was resuspended in pronase-CHAPS buffer (100 mM BTP pH 7.8, 100 mM CaCl_2_, with 0.5% w/v CHAPS (Anatrace)). Subsequently, pronase (Roche) was added at approximately 10-times the weight of the RGR substrate. Then the digestion mixture was incubated at 8°C–10°C for 24 h with gentle agitation using a shaker. The digest was desalted using a BioPureSPN C18 spin column. The column was washed with 30% ACN to remove CHAPS, and the N^ε^-retinyl-Lys products were eluted with 50% ACN. The N^ε^-retinyl-Lys products in the eluent were separated using a Vanquish Flex HPLC system with an XBridge C18 column and a 16-min gradient of 30%–38% ACN in water with 0.1% FA at a flow rate of 0.3 mL/min. N^ε^-retinyl-Lys products were detected by HPLC absorbance at 330 nm and identified by MS/MS with CID fragmentation using the LTQ XL. The isomeric identities of the retinyl moieties bound to Lys were distinguished by their absorbance spectrum, using the 1260 Infinity HPLC system with the same chromatographic conditions.

#### Monitoring hydrolysis of RGR*-11-*cis*-retinylidene

The following method was adapted from Hong et al.^19^ to track hydrolysis of the 11-*cis*-retinylidene adduct of RGR* in RPE microsomes. The RPE microsomes were incubated in the dark room under dim red light for 15 min with 20 μM all-*trans*-retinal to regenerate RGR. Next, samples were kept at 0°C to limit hydrolysis and exposed for 10 s to 530-nm light from a fiber-coupled LED set to an intensity of 125 μW at 0°C to determine the maximal yield of photoisomerization of the all-*trans*-retinylidene adduct of RGR to the 11-*cis*-retinylidene adduct of RGR*. For analysis of hydrolysis, the same photic conditions were repeated at 20°C with further incubation for 0, 30, and 60 s in the dark. NaBH_4_ in *i*PrOH was added to each sample after completion of the particular experimental condition: dark; light for 10 s at 0°C; light for 10 s at 20°C; light for 10 s at 20°C, followed by 30 s in the dark; and light for 10 s at 20C, followed by 60 s in the dark. The resultant protein precipitates were digested with pronase to produce N^ε^-retinyl-Lys products for chromatographic analysis by the Agilent 1260 Infinity HPLC system, as described above. The retinoid content of the alcohol-solubilized supernatant was analyzed, using the Agilent 1260 Infinity HPLC system with an XBridge C18 column and a mobile phase composition of MeOH with 0.1% FA (solvent A) and water with 0.1% FA (solvent B). Retinoids (namely, 11-*cis*- and all-*trans*-retinol, the NaBH_4_ reduction products of the respective aldehydes, were separated using a 15-min gradient of 80%–100% solvent B, followed by 20 min of 100% solvent B.

To determine the kinetics of hydrolysis of 11-*cis*-retinylidene-RGR*, we calculated the time-dependent mole fraction: moles of 11-*cis*-retinal bound to opsin as Schiff base, divided by the sum of opsin-bound 11-*cis*-retinal plus free 11-*cis*-retinal (released by hydrolysis of RGR*). The molar amount of 11-*cis*-retinal in the RPE microsomes was determined by measuring 11-*cis*-retinol in the alcohol soluble supernatant after NaBH_4_/*i*PrOH-treatment, using a 11-*cis*-retinol standard curve. Endogenous amounts of 11-*cis*-retinol or retinal were determined by measuring 11-*cis*-retinol after NaBH_4_/*i*PrOH-treatment of RPE microsomes without added all-*trans*-retinal. The molar amount of 11-*cis*-retinal generated from hydrolysis of RGR* was determined by the difference between the total 11-*cis*-retinol at each time point and the endogenous amount of 11-*cis*-retinol. The molar amount of 11-*cis*-retinylidene bound to RGR* was quantified, using a N^ε^-11-*cis*-retinyl-Lys standard curve. The mole fraction of 11-*cis*-retinylidene bound to RGR* over the total retinal (opsin-bound 11-*cis*-retinal plus free 11-*cis*-retinal generated by RGR* hydrolysis) was plotted for each time-point, generating a curve which was consistent with pseudo first order decay kinetics.

#### Single-cell RNA-seq data analysis

Mouse retina scRNA-seq was obtained from Campello, Brooks et al. (Manuscript in preparation), Gene Expression Omnibus (GEO) accession number GSE230049 and Luu et al.,^27^ GEO accession number GSE208760. Human^22^ and macaque^21^ scRNA-seq data was obtained via Broad Institute’s Single Cell Portal, accession numbers SCP839 and SCP212, respectively. Further analyses were performed using R Project for Statistical Computing (The R Foundation), utilizing Custom R scripts deposited in Github. Differentially expressed gene analysis in RGR positive versus RGR negative Müller cells was performed using the Wilcoxon Ranked Sum test of FindMarkers function in Seurat.^56^ Plots of scRNA data were prepared using VlnPlot and DotPlot in Seurat. DAVID was used to test the enrichment of GO terms from lists of species-specific DEGs (*P*-adj <0.01)^53,54^

#### Genotyping

Genomic DNA was derived from ear punches by incubation in 150 μL of DirectPCR (tail) lysis solution (Viagen Biotech) with 4 mg/mL proteinase K (Viagen Biotech): at 55°C overnight followed by 85°C for 1 h. Samples were centrifuged at 1,000*g* for 3 min and the supernatant (DNA extract) at 25-fold dilution was used in PCR genotyping reactions with GoTaq Green Master Mix (Promega). For *Rgr*^S^ animals, primer pairs a/b (0.67 kB product) and a/d (0.33 kB product; Table S2) were used to confirm the presence of the *Rgr*^S^ and *Rgr*^+^ alleles, respectively. Genotyping of other alleles followed the established protocols: *Pdeb*^rd1,58^ *Crb1*^rd8,59^ *Gnat1*^−^,^60^ *Rpe65*^CreERT2,32^ and *Glast-Cre*^ERT2,33^ using the appropriate primers, as listed in Table S2. The *Rpe65* M/L450 genotyping^61^ involved digestion of the PCR products with MwoI restriction enzyme (Thermo Fisher Scientific). All reactions were analyzed by agarose gel electrophoresis (Figure S6).

#### Cre induction

Cre recombinase activity in *Rpe65*^CreERT2^ and *Glast-Cre*^ERT2^ mice was induced by feeding the 2-month animals with tamoxifen-supplemented chow (250 mg/kg, Envigo TD.130856) for 3 weeks (+tamoxifen). Control animals were simultaneously fed with regular chow (− tamoxifen). Animals in each group (same genotype, age, and sex) were randomly assigned to either + or − tamoxifen cohort. Animals were fed with regular chow for one additional week atfter the 3-week period before the experimental procedures were performed.

#### Immunoblotting

The enucleated eyes were dissected to separate the neural retina and posterior eyecup (RPE, choroid, sclera). Each sample involved tissue pooling from three animals, biological replicates involved the use of animals from different litters. Tissue samples were incubated for 30 min at 4°C with shaking in 50 mM Tris, pH 7.4, 150 mM NaCl, 1% sodium dodecyl sulfate (Thermo Fisher Scientific), 5 mM tris-(2-carboxyethyl)phosphine (Biosynth), supplemented with Complete Ultra protease inhibitor cocktail (Roche). Samples were further sonicated for 1 min on ice and centrifuged at 21,000*g* for 15 min at 4°C. Supernatants mixed with 4x Laemmli Sample Buffer (Bio-Rad) were separated on 4–20% polyacrylamide gradient gels (Bio-Rad) and transferred onto 0.2-μm nitrocellulose membranes (Bio-Rad), using an eBlot L1 wet-transfer system (Genscript), according to the manufacturer’s recommendations. Membranes were incubated in the blocking buffer: 5% (w/v) nonfat milk (Research Products International) in TBS-T: Tris-buffered saline (TBS) with 0.1% Tween 20 (MilliporeSigma), for 1 h at room temperature (RT); and subsequently incubated overnight at 4°C in the blocking buffer with the appropriate primary antibodies, as listed in Table S3. After 3 washes with TBS-T, membranes were incubated in blocking buffer with secondary antibodies (Table S3) for 1 h at RT, and subjected to three more washes with TBS-T. For detection of the horseradish peroxidase-conjugated antibody, SuperSignal West Pico-Plus Chemiluminescent Substrate (Thermo Fisher Scientific) was used. Band visualization was performed using the ChemiDoc imaging system (Bio-Rad), and Image Lab software (Bio-Rad) was used for protein quantification. Band intensities were normalized to the respective GAPDH band intensities (internal standard).

#### In vivo retina imaging

Following pupil dilation with 1% tropicamide (Akorn), mice were anesthetized with an IP injection of ketamine/xylazine solution (100/10 mg/kg). A Bioptigen *in vivo* spectral-domain OCT device (Leica Microsystems) was used to perform rectangular scans at a rate of 1200 A-scans/B-scan. For each eye, an average of five repeated B-scans centered on the optic nerve head (ONH) and acquired at 0 and 90° were used for analysis. Retinal ONL thickness was measured 500 μm away from the ONH in four retinal quadrants (superior, inferior, nasal, temporal), and further averaged to give an overall value per eye. SLO was performed using a retinal angiograph Spectralis (Heidelberg Engineering) in the autofluorescence mode, and acquired images were analyzed qualitatively.

#### Histology

The enucleated mouse eyes were kept in Hartman’s fixative (MilliporeSigma) for 24 h at RT, transferred to 70% ethanol, and shipped to HistoWiz Inc for further processing according to their Standard Operating Procedure and fully automated workflow. Sagittally cut 6-μm paraffin sections spanning the ONH were stained with hematoxylin and eosin (H&E) and imaged with light microscopy with Aperio AT2 (Leica Biosystems) or BZ-X800 (Keyence) instruments. Manual counting of photoreceptor nuclei per row, every 300 μm starting from the edge of the ONH along both superior and inferior directions, was performed using QuPath software.^62^ Average values of ONL nuclei counts for each animal group represent data obtained from 5 eyes.

#### Immunohistochemistry

Enucleated mouse eyes were rinsed in PBS and incubated for 10 min at RT in freshly prepared fixing solution: PBS with 4% paraformaldehyde (Electron Microscopy Sciences). Following dissection along the posterior margin of the limbus, lens and vitreous were removed and the remaining eyecups were further incubated in the fixing solution for 20 min at RT. Eyecups were then rinsed in PBS and subjected to three brief washes in 5% (w/v) sucrose (MilliporeSigma) in PBS, two 30 min incubations each in the 10% (w/v) and 20% (w/v) sucrose-PBS solutions, and an overnight incubation at 4°C in the embedding medium: 2:1 (v/v) mixture of 20% (w/v) sucrose solution in PBS and Tissue-Tek O.C.T. Compound (Sakura). Following the embedding and freezing on dry ice, the eyecups were cut into 10 μm-thick sagittal sections with a CM1850 cryostat-microtome (Leica Biosystems), placed on glass slides, and stored at −80°C until needed. Defrosted retinal sections were rehydrated with PBS for 1 h at RT, then incubated for 1 h at RT in the blocking buffer: PBS with 3% (w/v) bovine serum albumin (MilliporeSigma), 3% normal donkey serum (MilliporeSigma), and 0.1% Triton X-100 (MilliporeSigma). Sections were subsequently incubated overnight at 4°C in the blocking buffer with the appropriate primary antibodies, as listed in Table S3. After three washes with PBS containing 0.1% Triton X-100 (PBS-T), the samples were incubated for 1 h at RT in blocking buffer with secondary antibodies (Table S3), followed by PBS with DAPI nuclear stain (Thermo Fisher Scientific) for 15 min at RT. Next, sections were washed three times with PBS-T and mounted with ProLong Glass Antifade medium (Thermo Fisher Scientific) for imaging. Fluorescence images were acquired using BZ-X800 (Keyence) and Elyra 7 (Zeiss) microscopes at low and high magnification, respectively. High magnification images were analyzed in ZEN Digital Imaging for Light Microscopy (Zeiss).

#### *In situ* hybridization

The enucleated mouse eyes were kept in Hartman’s fixative overnight at RT and transferred to 30% sucrose in PBS for 24 h incubation at 4°C, prior to embedding in O.C.T. compound for cryosectioning. Slides were processed for *in situ* hybridization using the RNAscope Multiplex Fluorescent Kit (Advanced Cell Diagnostics), according to manufacturer’s instructions, using custom probes targeting *Rgr* (Mm-Rgr-01-C1) and *Rlbp1* (Mm-Rlbp1-C2) transcripts (Table S2). Fluorescence images were obtained on an Elyra 7 (Zeiss) confocal microscope and analyzed in ZEN Digital Imaging for Light Microscopy (Zeiss).

#### In vivo ERG

Mice, dark-adapted overnight, were anesthetized with an IP injection of a mixture of ketamine/xylazine solution (100/4 mg/kg), and their pupils were dilated with a drop of 1% atropine sulfate. The temperature of each mouse’s body was maintained at 37°C with a heating pad. ERG responses were measured from both eyes by contact corneal electrodes held in place by a drop of Gonak solution (Akorn). Full-field ERGs were recorded with the UTAS BigShot apparatus (LKC Technologies), using Ganzfeld-derived test flashes of calibrated green 530-nm LED light (within a range from 0.24 cd s m^−2^ to 23.5 cd s m^−2^). M-cone ERG b-wave flash sensitivity $\left(S_{f}\right)$ was calculated from the linear part of the intensity-response, as follows:

$$S_{f}=A/\left(A_{max}\cdot I\right),$$

where A is the cone b-wave dim flash response amplitude (in μV), $A_{max}$ is the maximal response amplitude for that retina (in μV), and I is the flash strength (in cd • s m^−2^). For each eye, sensitivity was first determined in the dark and normalized to the maximum cone ERG b-wave amplitude obtained with the brightest white light stimulus delivered by the xenon flash tube (700 cd s m^−2^). Then, bright green background Ganzfeld illumination (300 cd m^−2^; estimated to bleach ~0.8% dark-adapted M-cone pigment sec^−1^) was applied continuously for 60 min while the M-cone b-wave $S_{f}$ change was monitored periodically. In the middle of the illumination period, an additional dose of anesthetic was applied (~1/2 of the initial dose), and a 1:1 mixture of PBS and Gonak solutions was gently applied to the eyes with a plastic syringe to protect them from drying, and to maintain electrode contacts. After the 60-min illumination period, the recovery of cone ERG b-wave $S_{f}$ was followed in darkness for up to 30 min.

Rod $S_{f}$ was calculated in an analogous way, except respective dim flash and maximal ERG a-wave amplitudes of *Gnat1*^+/+^ mice were used. In this case, green background Ganzfeld illumination (300 cd m^−2^) was applied continuously for 30 min, over which rods were not responsive to test flashes due to their physiological saturation. After the 30-min illumination, the recovery of rod ERG a-wave $S_{f}$ was followed in darkness for up to 90 min.

In a subset of experiments, the background illumination step was omitted, and cone $S_{f}$ recovery was monitored after acute >90% bleaching of cone pigment by a 35-s exposure to 520-nm bright LED light focused at the surface of the mouse eye cornea was applied to dark-adapted animals. The bleaching fraction was estimated by the following formula:

F=1−exp(−I⋅P⋅t)

where F is the fraction of pigment bleached, t is the duration of the light exposure (in seconds), I is the bleaching light intensity of 520-nm LED light (1.× 3 10^8^ photons μm^−2^ s^−1^), and P is the photosensitivity of mouse M-cones at the wavelength of peak absorbance (7.5 × 10^−9^ μm^−2^, adopted from^63^).

#### Retinoids extraction and analysis

All animals were dark-adapted overnight. For light exposure experiments, pupils were dilated with 1% tropicamide and mice were anesthetized with an IP injection of ketamine/xylazine solution (100/10 mg/kg). A green (530 nm) mounted LED equipped with collimation optics (Thorlabs) was placed at approx. 15 mm distance across each eye and set to an intensity of 1 mW using a DC4100 LED driver (Thorlabs). Light power was measured with the optical power meter PM100D equipped with S120VC light sensor (Thorlabs). The temperature of each mouse’s body was maintained at 37°C with a heating pad. Enucleated eyes were flash frozen (2 per sample) and stored at −80°C for further use. Under dim red light, eyes were placed in a Dounce homogenizer (Kimble Kontes) in 500 μL of cold homogenization buffer (20 mM Na phosphate pH 8.0, 100 mM hydroxylamine) with 500 μL cold MeOH. Retinoids were extracted from the homogenate by mixing with 2 mL of 5 M NaCl and 4 mL of methyl-*tert*-butyl-ether (Fisher Scientific). The organic layer was dried *in vacuo* and reconstituted in 300 μL of heptane (MilliporeSigma). The resulting suspension was centrifuged at 20,000*g*, and 100 μL of extract was injected for normal-phase HPLC analysis (Zorbax Rx-Sil 5 μm, 4.6 × 250 mm; Agilent Technologies) in a stepwise gradient of ethyl acetate in hexanes (Fisher Scientific): 0–17 min, 0.5%; 17.01–50 min, 10%; at a flow rate of 1.4 mL min^−1^, using the 1260 Infinity HPLC system. Retinoids were detected by monitoring absorbance at 325 nm for retinyl esters and retinol and at 360 nm for retinyloximes. Retinoids were quantified based on a standard curve relating the chromatographic peak area and the molar amount of each retinoid standard.

#### Two-photon excited fluorescence imaging of RPE flatmounts

Enucleated mouse eyes from dark-adapted (overnight) or daylight-adapted (≥ 1 h) animals were rinsed in PBS and incubated for 5 min at RT in freshly prepared fixing solution: PBS with 4% paraformaldehyde. Dark-adapted eyes were processed under dim red light and dissected using night vision goggles. Following dissection along the posterior margin of the limbus, lens and vitreous were removed and four radial cuts were made toward the optic nerve to flatten the remaining eyecups on glass slides. The samples were then mounted with Vectashield medium (Vector Laboratories) for imaging. To image tissue from pigmented animals, a customized TCS SP8 MP multiphoton imaging system (Leica Microsystems) with a pulse selection system delivering 75 fsec pulses at 8 MHz was used.^29^ Phasor analyses of FLIM data in Application Suite X (Leica Microsystems) were used to confirm identity of fluorescing compounds and determine their subcellular distribution.^29^ ImageJ analyze-particles software (NIH) was used to quantify the area occupied by retinosomes.

### QUANTIFICATION AND STATISTICAL ANALYSIS

Each experiment was reproduced at least three times and all measurements were taken from distinct biological samples. Exact numbers of biological replicates (n) and what they represent are reported in the figure legends. No statistical methods were applied to predetermine sample size. The experiments were not randomized, and the investigators were not blinded to allocation prior to data analysis. All healthy animals were included in the study. Quantitative data are expressed as mean ± SD or mean ± SEM as indicated. All statistical comparisons were made using Prism 9 software (GraphPad). p values less than 0.05 were considered statistically significant. Data were tested for normal distribution using Shapiro-Wilk test followed by evaluation using an appropriate test. Two-tailed Student’s t-test was performed for comparison between two groups of samples, Holm-Šídák correction was applied where multiple comparisons were performed. For gene-enrichment analysis, *P*-adj values were calculated using Benjamini-Hochberg method. For ERG recording analysis, two-way or three-way repeated measures ANOVA tests were used to analyze the effects of the independent factors defined for each experiment. *Post-hoc* tests to ANOVA utilized the Holm-Šídák approach. Details of the statistical analysis applied for particular comparisons are indicated in the figure legends.

## Supplementary Material

1

## Figures and Tables

**KEY RESOURCES TABLE:** 

REAGENT or RESOURCE	SOURCE	IDENTIFIER

Antibodies		

anti-Cralbp	Thermo Fisher Scientific	Cat#MA1-813; RRID: AB_2178528
anti-Gapdh	Proteintech	Cat#10494-1-AP; RRID: AB_2263076
anti-Lrat	Generated in-house^50^	N/A
anti-Rdh5	Antibodies-online	Cat#ABIN7254060
anti-Rdh8	Generated in-house^51^	N/A
anti-Rdh10	Antibodies-online	Cat#ABIN7118460
anti-Rdh12	Antibodies-online	Cat#ABIN7167836
anti-Rgr	Antibodies-online	Cat#ABIN7271760
anti-Rpe65	Generated in-house^52^	Clone: KPSA1
anti-Stra6	Thermo Fisher Scientific	Cat#PA5-100341; RRID: AB_2849855
anti-mouse IgG (HRP)	Promega	Cat#W4021; RRID: AB_430834
anti-mouse IgG (AF488)	Thermo Fisher Scientific	Cat#A32766; RRID: AB_2762823
anti-rabbit IgG (HRP)	Cell Signaling Technology	Cat#7074S; RRID: AB_2099233
anti-rabbit IgG (ID800CR)	LI-COR Biosciences	Cat#926-32211; RRID: AB_621843
anti-rabbit IgG (AF647)	Abcam	Cat#Ab150075; RRID: AB_2752244

Chemicals, peptides, and recombinant proteins		

Acetonitrile	Fisher Scientific	Cat#A955-4
all-frans-Retinal	MilliporeSigma	Cat#R2500
Atropine sulfate, 1%	Akorn	NDC: 17478-215-15
Bovine serum albumin	MilliporeSigma	Cat#A7030-100G
CHAPS	Anatrace	Cat#C316S
DAPI, 1 mg/mL	Thermo Fisher Scientific	Cat#62248
Formic acid	Fisher Scientific	Cat#A117-50
Gonak solution	Akorn	NDC: 17478-064-12
Hartman’s fixative	MilliporeSigma	Cat#H0290-1GAL
Heptane	MilliporeSigma	Cat#HX0078-6
Hexanes	Fisher Scientific	Cat#H302-4
Hydroxylamine	MilliporeSigma	Cat#159417-100G
Isopropanol	Fisher Scientific	Cat#A461-4
Ketamine, 100 mg/mL	Dechra Veterinary Products	NDC: 11695-0701-1
Methanol	Fisher Scientific	Cat#A456-4
Methyl-*tert*-butyl-ether	Fisher Scientific	Cat#E127-4
Milk, nonfat dry	Research Products International	Cat#M17200.100.0
Mwol restriction enzyme	Thermo Fisher Scientific	Cat#ER1732
Normal donkey serum	MilliporeSigma	Cat#S30-100ML
Paraformaldehyde, 16%	Electron Microscopy Sciences	Cat#15710
Pronase	Roche	Cat#10165921001
Protease inhibitor cocktail, Complete Ultra	Roche	Cat#05892953001
Proteinase K solution, 20 mg/mL	Viagen Biotech	Cat#501-PK
Sodium borohydrate	Fisher Scientific	Cat# AC200050250
Sodium dodecyl sulfate	Thermo Fisher Scientific	Cat#J18220
Tissue-tek O.C.T. compound	Sakura	Cat#4583
Tris-(2-carboxyethyl) phosphine hydrochloride	Biosynth	Cat#C-1818
Triton X-100	MilliporeSigma	Cat#X100-500ML
Tween 20	MilliporeSigma	Cat#P9416-50ML
Tropicamide, 1%	Akorn	NDC: 17478-102-12
Xylazine, 100 mg/mL (Rompun)	Dechra Veterinary Products	NDC: 17033-099-05

Critical commercial assays		

Antifade medium, ProLong Glass	Thermo Fisher Scientific	Cat#P36980
Antifade medium, Vectashield	Vector Laboratories	Cat#H-1000-10
DirectPCR (tail) lysis solution	Viagen Biotech	Cat#102-T
eBlot L1 transfer sandwich	Genscript	Cat#L00724
GoTaq Green Master Mix	Promega	Cat#M7823
Hematoxylin and Eosin staining	HistoWiz	Cat#1HE
Laemmli Sample Buffer, 4x	Bio-Rad	Cat#1610747
Polyacrylamide gels, Mini-PROTEAN TGX 4–20%	Bio-Rad	Cat#4561096
RNAscope Multiplex Fluorescent Reagent Kit v2	Advanced Cell Diagnostics	Cat#323100
SuperSignal West Pico PLUS Chemiluminescent Substrate	Thermo Fisher Scientific	Cat#34577

Deposited data		

Human scRNA-seq	(Yan et al.)^22^	Broad Institute Single Cell Portal: SCP839
Macaque scRNA-seq	(Peng et al.)^21^	Broad Institute Single Cell Portal: SCP212
Mouse scRNA-seq	(Campello, Brooks et al.; manuscript in preparation)	GEO: GSE230049
Mouse scRNA-seq	(Luu et al.)^27^	GEO: GSE208760

Experimental models: Organisms/strains		

Mouse: *Rgr^S^* : *Rgr^Stop^*	This paper, deposited in Jackson Laboratory	Strain #: 038172
Mouse: wild type: C57BL/6J	Jackson Laboratory	Strain #: 000664; RRID: IMSR_JAX:000664
Mouse: *Gnat1^−^*	Janis Lem (Calvert et al.)^31^	N/A
Mouse: *Rpe65*^CreERT2^: C57BL/6-*Rpe65^tm1.1(cre,ERT2)Kser^*/J)	Jackson Laboratory	Strain #: 035973; RRID: IMSR_JAX:035973
Mouse: *Glast-Cre*^ER12^: Tg(Slc1a3-cre/ERT)1Nat/J	Jackson Laboratory	Strain #: 012586; RRID: IMSR_JAX:012586

Oligonucleotides		

ISH Probes	Table S2	N/A
Primers for genotyping	Table S2	N/A

Software and algorithms		

Custom R scripts	This paper	https://doi.org/10.5281/zenodo.8140173 https://github.com/NEI-NNRL/2023_RGR_Muller_glia
DAVID	NIH (Huang et al.),^53^ (Huang et al.)^54^	RRID: SCR_001881 https://david.ncifcrf.gov/
GraphPad Prism 9	GraphPad	RRID: SCR_002798 http://www.graphpad.com/
Image Lab	Bio-Rad	RRID: SCR_014210 http://www.bio-rad.com/en-us/sku/1709690-image-lab-software
ImageJ	(Schneider et al.)^55^	RRID: SCR_003070 https://imagej.net/
Leica Application Suite X	Leica Microsystems	RRID: SCR_013673 https://www.leica-microsystems.com/products/microscope-software/details/product/leica-las-x-ls/
QuPath	Queens University Belfast	RRID: SCR_018257 https://qupath.github.io/
R Project for Statistical Computing	The R Foundation	RRID: SCR_001905 http://www.r-project.org/
Seurat v4.3.0	(Satija et al.)^56^	RRID: SCR_016341 https://satijalab.org/seurat/get_started.html
ZEN Digital Imaging for Light Microscopy	Zeiss	RRID: SCR_013672 http://www.zeiss.com/microscopy/en_us/products/microscope-software/zen.html#introduction

Other		

Cryostat-microtome	Leica Biosystems	Cat#CM1850
Desalting column, BioPure SPN Midi, TARGAC18	The Nest Group	Cat#HEM S18R
Dounce homogenizer, size 21, 3 mL	Kimble Kontes	Cat#885460-0021
eBlot L1 wet-transfer system	Genscript	Cat#L00686
Fluorescence microscope	Keyence	Cat#BZ-X800E
HPLC column, XBridge BEH C18	Waters	Cat#186003022
HPLC column, Zorbax Rx-Sil 5 mm	Agilent Technologies	Cat#880975-901
HPLC system, Vanquish Flex Binary	Thermo Fisher Scientific	Cat#IQLAAAGABHFAPUMBJC
HPLC system, 1260 Infinity	Agilent Technologies	N/A
Imaging system, ChemiDoc MP	Bio-Rad	N/A
LED, 530 nm fiber-coupled	Thorlabs	Cat#M530F2
LED, 530 nm mounted	Thorlabs	Cat#M530L3
LED Driver, 4-Channel Thorlabs	Cat#DC4100
LED Driver, T-Cube	Thorlabs	Cat#LEDD1B
Nitrocellulose Membrane, 0.2 mm	Bio-Rad	Cat#1620112
Mass spectrometer, LTQ XL Linear Ion Trap	Thermo Fisher Scientific	Cat#IQLAAEGAAVFACZMAIK
Microscope, confocal Elyra 7	Zeiss	N/A
Microscope, fluorescence	Keyence	Cat#BZ-X800E
Microscope, multiphoton TCS SP8 MP	Leica Microsystems	N/A
Optical Power and Energy Meter	Thorlabs	Cat#PM100D
Photodiode Power Sensor	Thorlabs	Cat#S120VC
Slide scanner, Aperio AT2	Leica Biosystems	N/A
SLO retinal angiograph, Spectralis	Heidelberg Engineering	N/A
Spectral-domain OCT, Bioptigen Envisu	Leica Microsystems	N/A
Tamoxifen diet for mice (250 mg/kg)	Envigo	Cat#TD.130856
Visual electrodiagnostic system, UTAS BigShot	LKC Technologies	N/A

## Highlights

- RGR supports rapid photoproduction of the visual chromophore
- A subpopulation of Müller glia exhibits specialization in supporting the photic visual cycle
- RPE and Müller glia RGR pools contribute to cone visual pigment recycling
- RGR accelerates rod dark adaptation upon the transition from bright light to darkness
