# Supplementary material for: Rapid RGR-dependent visual pigment recycling is mediated by the RPE and specialized Müller glia
Source: Cell Rep. Author manuscript; Available in PMC 2023 Sep 27. (PMC10530494; doi:10.1016/j.celrep.2023.112982)
Supplement: 1 [file NIHMS1928285-supplement-1.pdf]

Cell Reports, Volume *42*

Supplemental information

Rapid RGR-dependent visual pigment recycling is mediated by the RPE and specialized Müller glia

Aleksander Tworak, Alexander V. Kolesnikov, John D. Hong, Elliot H. Choi, Jennings C. Luu, Grazyna Palczewska, Zhiqian Dong, Dominik Lewandowski, Matthew J. Brooks, Laura Campello, Anand Swaroop, Philip D. Kiser, Vladimir J. Kefalov, and Krzysztof Palczewski

SUPPLEMENTARY MATERIALS

Contents

Figure S1. LC-MS/MS analysis of proteinase K digest of bRGR from RPE microsomal membranes.

Figure S2. LC-MS/MS analysis of pronase digest of bRGR from RPE microsomal membranes.

Figure S3. Expression of chromophore recycling-associated proteins in the mouse, macaque, and human retina.

Figure S4. *In situ* hybridization using anti-Rgr and anti-Rlbp1 probes on cryosections from WT, KO, RPE-Cre, MG-Cre, and 2-Cre animals after tamoxifen treatment.

Figure S5. Comparison of the cone function in steady background light, and subsequent dark adaptation in the absence of tamoxifen in the three Cre-driver lines.

Figure S6. Genotyping results for Rgr^{S} (A), *Pdeb*^{rd1} (B), *Crb1*^{rd8} (C), *Gnat1*$^{-}$ (D), *Rpe65*^{CreERT2} (E), *Glast-Cre*^{ERT2} (F), and *Rpe65* M/L450 (G) variants.

Table S1. ANOVA statistics for mouse group comparisons presented in Figures 6, 7, and 8.

Table S2. List of oligonucleotides used in the study. Related to STAR Methods.

Table S3. List of antibodies and their dilutions used in the study. Related to STAR Methods.

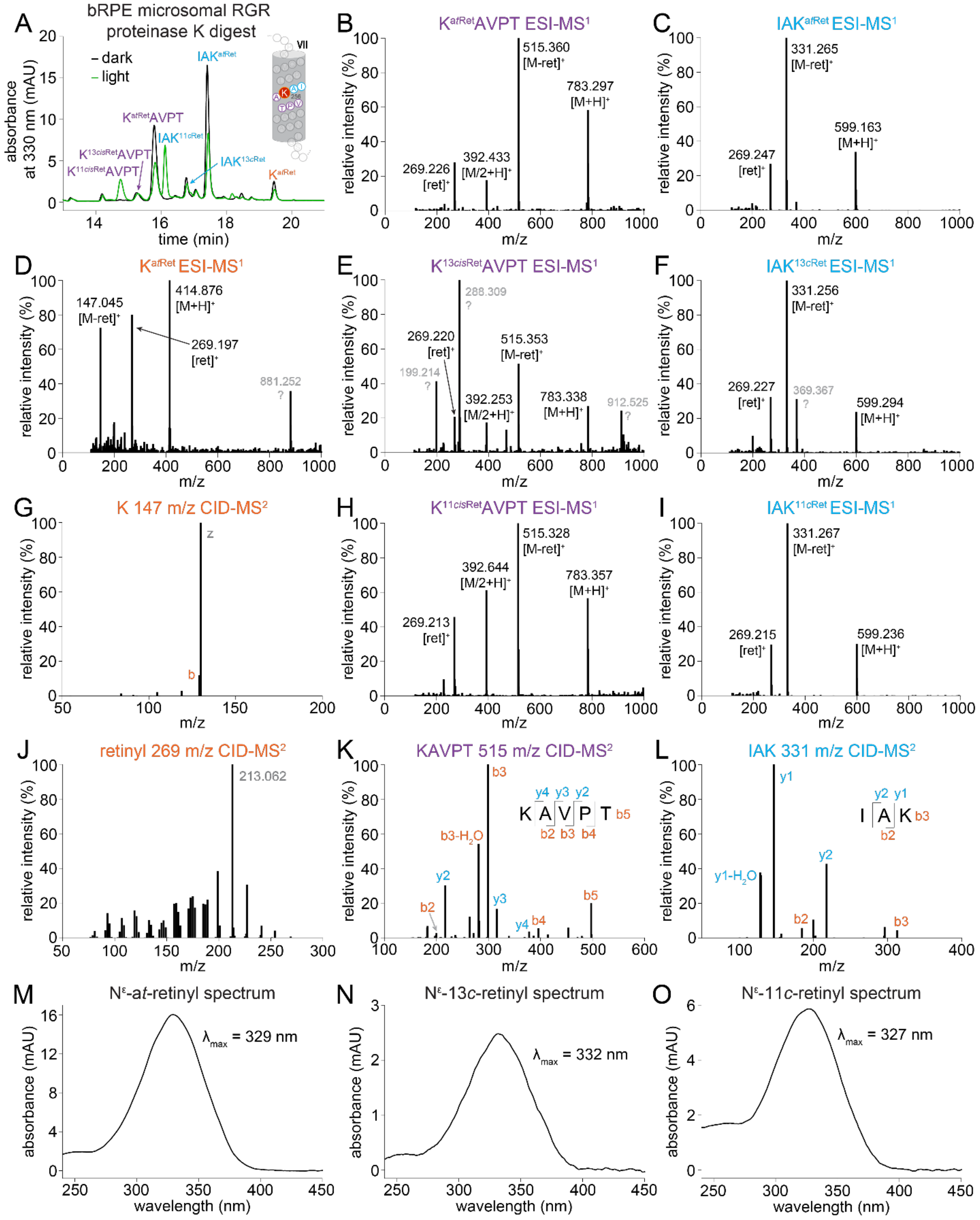

Figure S1. LC-MS/MS analysis of proteinase K digest of bRGR from RPE microsomal membranes. Related to Figure 1.

Panel A shows chromatographic separation and mass spectroscopic identification of N^{ε}-retinyl-peptides from proteinase K digestion of bRGR from $NaBH_4$/*i*PrOH-treated RPE microsomes, before and after light exposure. N^{ε}-retinyl-peptide products correspond to the all-*trans*-retinylidene Schiff base adduct of Lys^{256} of bRGR.

Panels B-F and H-I show the ESI-MS[1] spectra of N^{ε}-retinyl-peptide products from digestion of bRGR; each spectrum shows the characteristic partial-source fragmentation pattern, cleaving the retinyl cation from the precursor N^{ε}-retinyl-peptide ion and producing a product peptide ion that can be sequenced by CID fragmentation, as shown in panels G, K, and L.

Panel J shows the characteristic CID fragmentation pattern of the retinyl cation producing a dominant 213 m/z signal.

Panels M-O show the absorption spectrum of each retinyl isomer present in the N^{ε}-retinyl-peptide products from the proteinase K digest. Thermal isomerization during the digestion resulted in the minor amount of the 13-*cis* isomer, as reported in Hong *et al*. [19]. All experiments were performed in n = 3 biological replicates and representative results are shown.

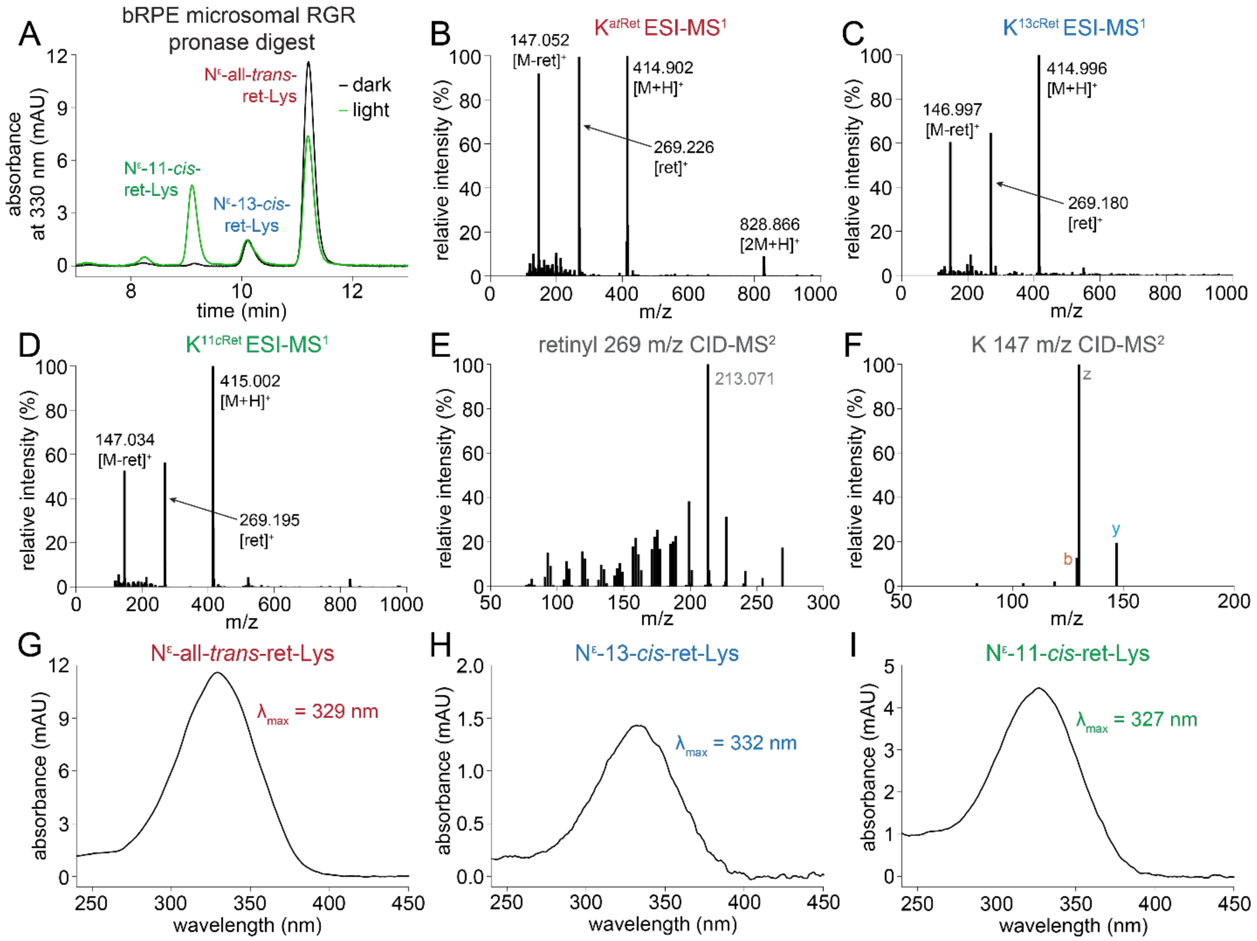

Figure S2. LC-MS/MS analysis of pronase digest of bRGR from RPE microsomal membranes. Related to Figure 1.

Panel A shows chromatographic separation and mass spectroscopic identification of N^{ε}-retinyl-Lys products from pronase digestion of bRGR from $NaBH_4$/*i*PrOH-treated RPE microsomes, before and after light exposure.

Panels B-D show the ESI-MS^1 spectra of N^{ε}-retinyl-Lys products from digestion of bRGR; each spectrum shows the characteristic partial source fragmentation pattern, cleaving the retinyl cation from the precursor N^{ε}-retinyl-Lys ion and producing a product Lys ion that can be confirmed by CID fragmentation, as shown in panel F.

Panel E shows the characteristic CID fragmentation pattern of the retinyl cation producing a dominant 213 m/z signal.

Panels G-I show the absorption spectrum of each retinyl isomer present in the N^{ε}-retinyl-Lys products from the pronase digest. Thermal isomerization during the digestion resulted in the minor amount of the 13-*cis* isomer, as reported in Hong *et al.* [19]. All experiments were performed in n = 3 biological replicates and representative results are shown.

A

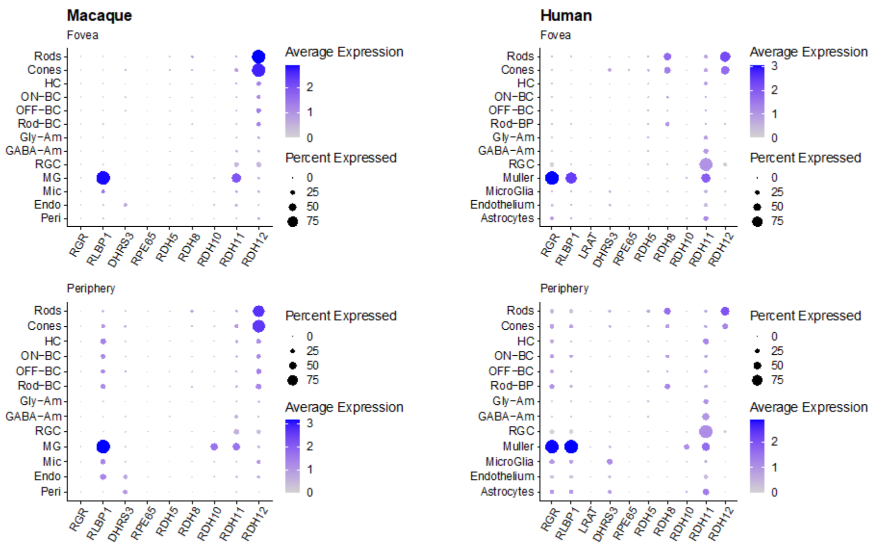

B

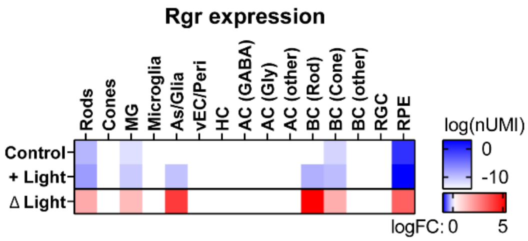

Figure S3. Expression of chromophore recycling-associated proteins in the macaque, and human retina. Related to Figure 2.

Panel A shows scRNA-seq-based expression levels of chromophore recycling-associated genes from different species. Dot-plots simultaneously indicate percentage of cells expressing the genes per cell type (dot size) and the expression levels within that group (color). Macaque and human data are separated based on retinal region from which the cells originated. Analyzed data involved n = 92,628 foveal and n = 73,053 peripheral macaque single cell profiles, as well as n = 55,736 foveal and n = 29,246 peripheral human single cell profiles.

Panel B shows scRNA-seq-based heatmap depicting average Rgr expression in n = 26,543 retinal cell types, quantified by normalized unique molecular identifier (nUMI) counts in control animals (control) and animals analyzed 1 day after bright-light exposure[27]. In the bottom row (Δ Light), log fold change (logFC) reveals Rgr upregulation 1 day after bright-light exposure relative to unexposed controls.

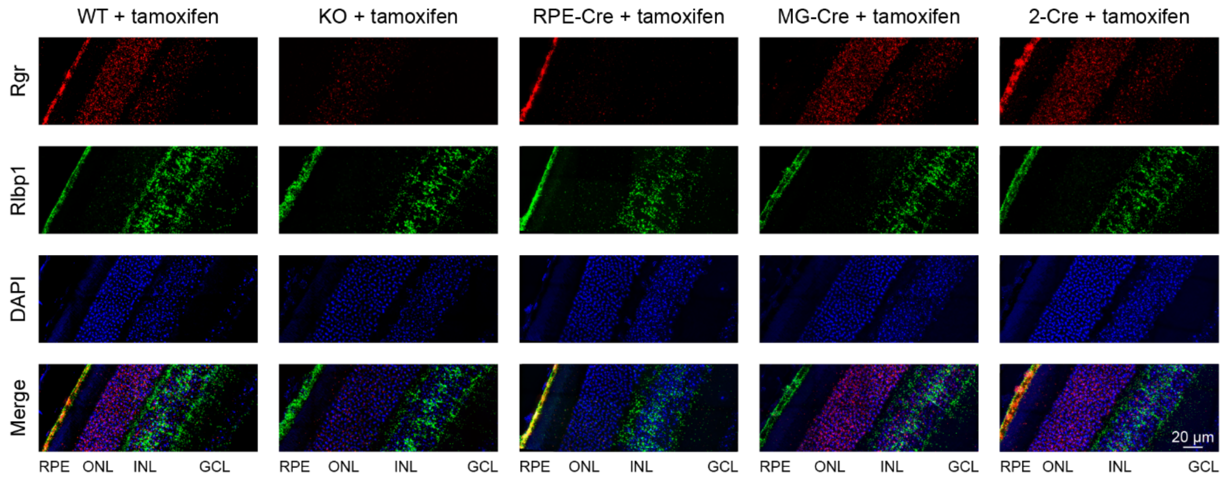

Figure S4. ***In situ*** **hybridization using anti-Rgr and anti-Rlbp1 probes on cryosections from WT, KO, RPE-Cre, MG-Cre, and 2-Cre animals after tamoxifen treatment**. **Related to Figure 5.**

Rgr expression, lost in the KO mouse line, is selectively restored in the RPE of RPE-Cre mice, Müller Glia of MG-Cre mice, and in both cell types of the 2-Cre mice. Genotype details for all mouse lines are indicated in Fig. 5A. Representative images are shown. Images were taken 250-500 µm from the ONH, scale bar: 20 µm.

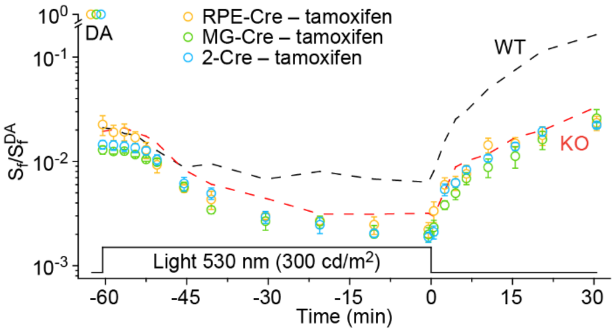

Figure S5. Comparison of the cone function in steady background light, and subsequent dark adaptation in the absence of tamoxifen in the three Cre-driver lines. Related to Figure 7.

The graph shows the changes in M-cone-driven ERG b-wave flash sensitivity (S_f) *in vivo* following illumination with green 530-nm Ganzfeld LED background light (300 cd m^{-2}, 60 min), and its subsequent recovery in the dark in tamoxifen-untreated RPE-Cre (n = 10 eyes), MG-Cre (n = 12 eyes), and 2-Cre (n = 12 eyes) mice. The dashed lines represent respective data for WT and KO animals, replotted from Figure 6C. Genotype details for all mouse lines are indicated in Fig. 5A. Data expressed as mean ± SEM (error bars are smaller than symbol size for most data points).

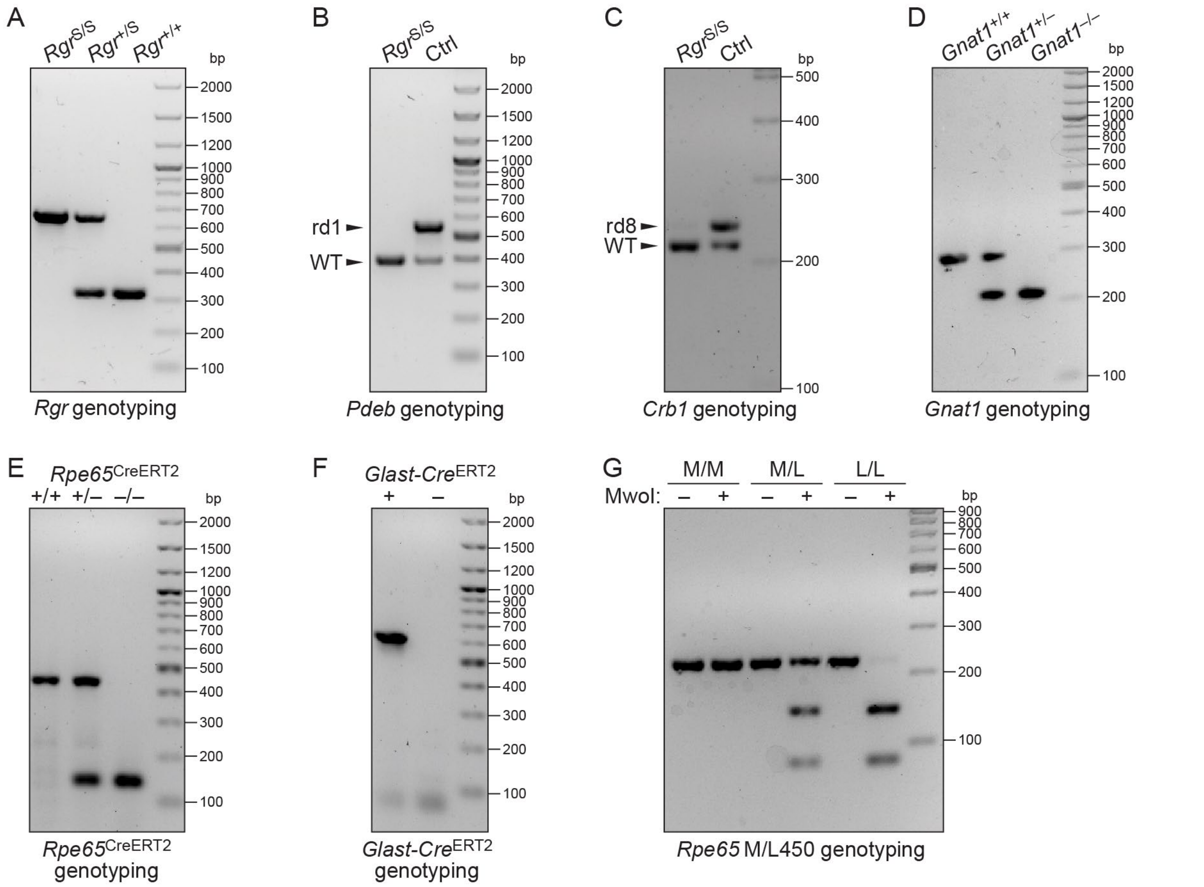

Figure S6. Genotyping results for Rgr^{S} (A), *Pde6b*^{rd1} (B), *Crb1*^{rd8} (C), *Gnat1*$^{-}$ (D), *Rpe65*^{CreERT2} (E), *Glast-Cre*^{ERT2} (F), and *Rpe65* M/L450 (G) variants. Related to STAR Methods.

Table S1. ANOVA statistics for mouse group comparisons presented in Figures 6, 7, and 8.

Factor	Sum of Squares	Degrees of Freedom	Mean Square	F ratio (DF numerator, DF denominator)	Significance (*P* value)
**Groups: WT -Tamoxifen (n=10), WT +Tamoxifen (n=14), KO -Tamoxifen (n=12), KO +Tamoxifen (n=14); 3-way ANOVA, matching by factor: Time point; Fig. 6C**					
Time point	0.5007	19	0.02635	F (1.773, 81.57) = 140.1	P<0.0001
Genotype	0.09149	1	0.09149	F (1, 46) = 89.83	P<0.0001
Tamoxifen treatment	0.0009876	1	0.0009876	F (1, 46) = 0.9697	P=0.3299
Time point × Genotype	0.2952	19	0.01554	F (19, 874) = 82.57	P<0.0001
Time point × Tamoxifen treatment	0.001346	19	7.082×10^{-5}	F (19, 874) = 0.3764	P=0.9931
Genotype × Tamoxifen treatment	2.485×10^{-5}	1	2.485×10^{-5}	F (1, 46) = 0.02440	P=0.8766
Time point × Genotype × Tamoxifen treatment	0.001047	19	5.511×10^{-5}	F (19, 874) = 0.2929	P=0.9987
Subject	0.04685	46	0.001018		
Residual	0.1644	874	0.0001881		
**Groups: RPE-Cre -Tamoxifen (n=10), RPE-Cre +Tamoxifen (n=16); 2-way ANOVA, matching by factor: Time point; Fig. 7A**					
Time × Mouse group	0.05288	19	0.002783	F (19, 456) = 6.887	P<0.0001
Time	0.1075	19	0.005658	F (1.123, 26.95) = 14.00	P=0.0006
Mouse group	0.01536	1	0.01536	F (1, 24) = 9.990	P=0.0042
Subject	0.03690	24	0.001537	F (24, 456) = 3.804	P<0.0001
Residual	0.1843	456	0.0004041		
**Groups: MG-Cre -Tamoxifen (n=12), MG-Cre +Tamoxifen (n=12); 2-way ANOVA, matching by factor: Time point; Fig. 7B**					
Time × Mouse group	0.01672	19	0.0008801	F (19, 418) = 5.329	P<0.0001
Time	0.05821	19	0.003064	F (1.364, 30.00) = 18.55	P<0.0001
Mouse group	0.006998	1	0.006998	F (1, 22) = 8.002	P=0.0098
Subject	0.01924	22	0.0008746	F (22, 418) = 5.295	P<0.0001
Residual	0.06904	418	0.0001652		
**Groups: 2-Cre -Tamoxifen (n=12), 2-Cre +Tamoxifen (n=12); 2-way ANOVA, matching by factor: Time point; Fig. 7C**					
Time × Mouse group	0.1443	19	0.007596	F (19, 418) = 35.11	P<0.0001
Time	0.2273	19	0.01196	F (1.583, 34.82) = 55.29	P<0.0001
Mouse group	0.05254	1	0.05254	F (1, 22) = 44.76	P<0.0001
Subject	0.02582	22	0.001174	F (22, 418) = 5.426	P<0.0001
Residual	0.09043	418	0.0002163		
**Groups: RPE-Cre +Tamoxifen (n=16), MG-Cre +Tamoxifen (n=12), 2-Cre +Tamoxifen (n=12); 2-way ANOVA, matching by factor: Time point; Fig. 7D**					
Time × Mouse group	0.06720	38	0.001768	F (38, 703) = 3.731	P<0.0001
Time	0.5535	19	0.02913	F (1.371, 50.71) = 61.46	P<0.0001
Mouse group	0.02475	2	0.01238	F (2, 37) = 6.255	P=0.0046
Subject	0.07322	37	0.001979	F (37, 703) = 4.175	P<0.0001
Residual	0.3332	703	0.0004740		
**Groups: $Rgr^{+/+}$ (n=12), $Rgr^{S/S}$ (n=10); 2-way ANOVA, matching by factor: Time point; Fig. 8A**					
Time × Genotype	0.3075	10	0.03075	F (10, 220) = 5.759	P<0.0001
Time	2.978	10	0.2978	F (2.720, 59.84) = 55.78	P<0.0001
Genotype	0.4569	1	0.4569	F (1, 22) = 10.23	P=0.0041
Subject	0.9823	22	0.04465	F (22, 220) = 8.364	P<0.0001
Residual	1.174	220	0.005339		
**Groups: $Rgr^{+/+}$ (n=12), $Rgr^{S/S}$ (n=10); 2-way ANOVA, matching by factor: Time point; Fig. 8C**					
Time × Genotype	0.02515	11	0.002286	F (11, 220) = 3.796	P<0.0001
Time	0.1016	11	0.009234	F (1.224, 24.48) = 15.33	P=0.0003
Genotype	0.02738	1	0.02738	F (1, 20) = 5.416	P=0.0306
Subject	0.1011	20	0.005056	F (20, 220) = 8.395	P<0.0001
Residual	0.1325	220	0.0006023		

Table S2. List of oligonucleotides used in the study. Related to STAR Methods.

Target gene	Application	Target allele	Oligo ID	Source	Sequence
Rgr	ISH	wt	1096471-C1	Advanced Cell Diagnostics	N/A
Rlbp1	ISH	wt	468161-C2	Advanced Cell Diagnostics	N/A
Rgr	*Rgr*^{S} validation, genotyping	wt	Rgr a	This paper	CAAGAGATACCTGCCTCAAGCTAGAC
Rgr	*Rgr*^{S} validation, genotyping	S	Rgr b	This paper	CCAGAGGCCACTTGTGTAGC
Rgr	*Rgr*^{S} validation, genotyping	S	Rgr c	This paper	GCATCGCCTTCTATCGCCTTCTTG
Rgr	*Rgr*^{S} validation, genotyping	wt	Rgr d	This paper	ACTGTGGAGGCCAGTGCATGG
Pde6b	*Pde6b*^{rd1} genotyping	wt, rd1	RD3	(Giménez, *et al.*)[54]	TGACAATTACTCCTTTTCCCTCAGTCTG
Pde6b	*Pde6b*^{rd1} genotyping	rd1	RD4	(Giménez, *et al.*)[54]	GTAAACAGCAAGAGGCTTTATTGGGAAC
Pde6b	*Pde6b*^{rd1} genotyping	wt	RD6	(Giménez, *et al.*)[54]	TACCCACCCTTCCTAATTTTTCTCAGC
Crb1	*Crb1*^{rd8} genotyping	wt	mCrb1-mF1	(Mehalow, *et al.*)[55]	GTGAAGACAGCTACAGTTCTGATC
Crb1	*Crb1*^{rd8} genotyping	rd8	mCrb1-mF2	(Mehalow, *et al.*)[55]	GCCCCTGTTTGCATGGAGGAAACTTGGAAGACA GCTACAGTTCTTCTG
Crb1	*Crb1*^{rd8} genotyping	wt, rd8	mCrb1-mR	(Mehalow, *et al.*)[55]	GCCCCATTTGCACACTGATGAC
Gnat1	*Gnat1*$^{-}$ genotyping	wt	GnatKO-F1	(Zhu, *et al.*)[56]	CGAGTTCATTGCCATCATCTACG
Gnat1	*Gnat1*$^{-}$ genotyping	wt	GnatKO-R1	(Zhu, *et al.*)[56]	ATACCCGAGTCCTTCCACAAGC
Gnat1	*Gnat1*$^{-}$ genotyping	ko	GnatKO-F2	(Zhu, *et al.*)[56]	GAGGATTGGGAAGACAATAGCAG
Gnat1	*Gnat1*$^{-}$ genotyping	ko	GnatKO-R2	(Zhu, *et al.*)[56]	CACCAGCACCATGTCGTAAG
Rpe65	*Rpe65*^{CreERT2} genotyping	wt, cre	RPE65_E14f	(Choi, *et al.*)[32]	CTTCCATGGACTGTTCAAAAGATCC
Rpe65	*Rpe65*^{CreERT2} genotyping	wt	RPE65_E14r	(Choi, *et al.*)[32]	AACTTCCAGGAGTAAGTTCTGTCC
Rpe65	*Rpe65*^{CreERT2} genotyping	cre	RPE65_CreERT 2r	(Choi, *et al.*)[32]	GCATAACCAGTGAAACAGCATTG
Rpe65	*Rpe65* M/L450 genotyping	M/L 450	RPE65f	(Lopes, *et al.*)[57]	GCATACGGACTTGGGTTGAATCAC
Rpe65	*Rpe65* M/L450 genotyping	M/L 450	RPE65r	(Lopes, *et al.*)[57]	GGTTGAGAAACAAAGATGGGTTCAG
***Glast-Cre*^{ERT2}**	*Glast-Cre*^{ERT2} genotyping	cre	10110	(Wang, *et al.*)[33]	ACAATCTGGCCTGCTACCAAAGC
***Glast-Cre*^{ERT2}**	*Glast-Cre*^{ERT2} genotyping	cre	10112	(Wang, *et al.*)[33]	CCAGTGAAACAGCATTGCTGTC

Table S3. List of antibodies and their dilutions used in the study. Related to STAR Methods. [a]

Target	Host	Source	Catalog/clone number	Dilution in IHC	Dilution in WB
**Primary antibodies**					
CRALBP	Mouse	Thermo Fisher Scientific	MA1-813	1:500	1:5000
GAPDH	Rabbit	Proteintech	10494-1-AP	-	1:5000
LRAT	Mouse	Generated in-house[60]	n/a	-	1:200
RDH5	Rabbit	Antibodies-online	ABIN7254060	-	1:250
RDH8	Mouse	Generated in-house[61]	n/a	-	1:100
RDH10	Rabbit	Antibodies-online	ABIN7118460	-	1:500
RDH12	Rabbit	Antibodies-online	ABIN7167836	-	1:250
RGR	Rabbit	Antibodies-online	ABIN7271760	1:200	1:500
RPE65	Mouse	Generated in-house[62]	KPSA1	-	1:200
STRA6	Rabbit	Thermo Fisher Scientific	PA5-100341	-	1:1000
**Secondary antibodies**					
Mouse IgG (HRP)	Goat	Promega	W4021	-	1:2500
Mouse IgG (AF488)	Donkey	Thermo Fisher Scientific	A32766	1:500	-
Rabbit IgG (HRP)	Goat	Cell Signaling Technology	7074S	-	1:2500
Rabbit IgG (ID800CR)	Goat	LI-COR Biosciences	926-32211	-	1:15000
Rabbit IgG (AF647)	Donkey	Abcam	Ab150075	1:500	-

[a]Abbreviations used: AF, Alexa Fluor; CRALBP, cellular retinaldehyde-binding protein; GAPDH, Glyceraldehyde-3-phosphate dehydrogenase; HRP, Horseradish peroxidase; ID, IRDye; IgG, Immunoglobulin G; IHC, Immunohistochemstry; LRAT, Lecithin retinol acyltransferase; RDH, Retinol dehydrogenase; RGR, Retinal G protein-coupled receptor; RPE65, Retinoid isomerase; STRA6, Receptor for retinol uptake STRA6; WB, Western blot.
